# Pioneering Investigation on the Larvicidal Mechanism and Chemical Profile of *Piper humillimum* C.DC. (Piperaceae) Essential Oil: Integrating *In Vivo*, *In Vitro*, and *In Silico* Models Against *Aedes aegypti* (Linnaeus, 1762) and *Anopheles darlingi* Root, 1926 (Culicidae)

**DOI:** 10.3390/molecules31111960

**Published:** 2026-06-04

**Authors:** André C. de Oliveira, Maria Luiza L. da Costa, Gabriel M. Marcusso, Rejane C. Simões, Raynner N. G. Serrão, Élder Augusto G. Figueira, Gilson S. de Lima, Aldenora dos S. Vasconcelos, Jéssica A. Marques, Hector H. F. Koolen, Felipe M. A. da Silva, Ingrity S. Costa Sá, Rita de C. Saraiva Nunomura, Sergio M. Nunomura, Rosemary A. Roque

**Affiliations:** 1 Laboratório de Controle Biológico e Biotecnologia da Malária e da Dengue, Coordenação da Sociedade, Ambiente e Saúde, Instituto Nacional de Pesquisas da Amazônia, Manaus 69067-375, AM, Brazil; 2 Programa de Pós-Graduação em Biodiversidade e Biotecnologia da Rede Bionorte, Centro Multiusuário para Análise de Fenômenos Biomédicos, Universidade do Estado do Amazonas, Manaus 69065-001, AM, Brazil; 3 Programa de Pós-Graduação em Inovação Farmacêutica, Faculdade de Ciências Farmacêuticas, Universidade Federal do Amazonas, Manaus 69077-000, AM, Brazil; 4 Instituto de Pesquisas Jardim Botânico do Rio de Janeiro, Rio de Janeiro 22460-000, RJ, Brazil; 5 Departamento de Vigilância Ambiental e Controle de Doenças, Fundação de Vigilância Em Saúde Do Amazonas, Dra Rosemary Costa Pinto, Manaus 69093-018, AM, Brazil; 6 Programa de Pós-Graduação em Medicina Tropical, Centro Multiusuário para Análise de Fenômenos Biomédicos, Universidade do Estado do Amazonas, Manaus 69065-001, AM, Brazil; j.araujo.9414@gmail.com; 7 Grupo de Pesquisa em Metabolômica e Espectrometria de Massas, Escola Superior de Ciências da Saúde, Centro Multiusuário para Análise de Fenômenos Biomédicos, Universidade do Estado do Amazonas, Manaus 69065-001, AM, Brazil; 8 Laboratório de Bioprospecção e Farmacologia de Produtos Naturais, Coordenação de Tecnologia e Inovação, Instituto Nacional de Pesquisas da Amazônia, Manaus 69080-971, AM, Brazil; 9 Instituto de Saúde e Biotecnologia, Campus Coari, Universidade Federal do Amazonas, Coari 69460-000, AM, Brazil; 10 Laboratório de Abertura de Amostras e Ensaios Químicos, Central Analítica, Centro de Apoio Multidisciplinar, Universidade Federal do Amazonas, Manaus 69080-900, AM, Brazil; ritasnunomura@gmail.com; 11 Laboratório de Princípios Ativos da Amazônia, Coordenação de Tecnologia e Inovação, Instituto Nacional de Pesquisas da Amazônia, Manaus 69067-375, AM, Brazil

**Keywords:** *Aedes*, *Anopheles*, *Piper*, mechanism, docking

## Abstract

*Aedes aegypti* and *Anopheles darlingi* represent health challenges due to synthetic insecticide resistance. Hence, the essential oil from *Piper humillimum* is an alternative for vector control. In this study, the essential oil (3.5 ± 0.4% yield) alongside germacrene D (61.51%) and δ-cadinene (17.46%) showed larvicidal activity (LC_50_ of 34.75 to 46.04 µg/mL), accompanied by an increase of hydrogen peroxide (H_2_O_2_) production (36.67 ± 1.52 to 81.33 ± 1.52 µmol H_2_O_2_ min^−1^ mg^−1^ protein), causing lipid (43.3 ± 6.02 to 81.67 ± 3.05 nmol malondialdehyde mg^−1^ protein) and protein damages (61.67 ± 6.80 to 83.00 ± 2.64 nmol carbonyls mg^−1^ protein). Further triggering an increase in superoxide dismutase (83.31 ± 6.80 to 95.00 ± 3.60 U mg^−1^ protein) and catalase (74.31 ± 7.02 to 82.09 ± 1.00 µmol H_2_O_2_ min^−1^ mg^−1^ protein) activities. In addition, mixed-function oxidases (61.17 ± 11.37 to 73.52 ± 6.42 nmol cyt c min^−1^ mg^−1^ protein), α- and β-esterase (38.41 ± 4.04 to 61.31 ± 9.29 µmol min^−1^ mg^−1^ protein) levels increased. Conversely, glutathione S-transferase (GST) (11.01 ± 2.00 to 9.67 ± 3.05 µmol min^−1^ mg^−1^ protein) and acetylcholinesterase (AChE) (14.33 ± 3.78 to 17.00 ± 1.00 μmol min^−1^ mg^−1^ protein) were inhibited, corroborated by molecular docking, with germacrene D and δ-cadinene showing binding energies of −7.9 and −7.9 kcal/mol, 1.63 and 1.94 Ki for AChE, while for GST were −6.4 and 6.6 kcal/mol, and 20.5 and 15.50 Ki, respectively. These results demonstrate that the essential oil from *P. humillimum* is a promising multi-target alternative for the control of the investigated vectors.

## 1. Introduction

Dengue is a major viral disease caused by the *Flavivirus* etiologic agent (*Orthoflaviviridae*), maintaining a high epidemic potential in several tropical countries, particularly in Brazil which remains a critical epicenter for the disease in the Americas, a situation underscored by alarming recent epidemiological data [1].

In 2025, Brazil reported 1,651,712 probable cases and 1821 deaths; furthermore, as of 23 March 2026, 146,193 cases and 36 deaths have already been registered [2]. These figures highlight the persistent severity of this public health crisis and the urgent need for effective vector control strategies [3].

Malaria is another disease with high epidemic potential in Brazil, being strictly endemic to the Amazon region, which accounts for 99% of all infections in the country [4]. In 2025, 117,712 cases were recorded, while as of 23 March 2026, 15,272 infections have already been registered, that notably, 93.89% of these cases were caused by *Plasmodium vivax*, whereas *P. falciparum* (Plasmodiidae) accounted for 6.11% [5].

This regional concentration, combined with the prevalence of diverse *Plasmodium* species, poses a continuous public health challenge, requiring precise and sustainable vector control strategies in the Amazonian basin [6].

DENV and *Plasmodium* parasites are transmitted in Brazil by their respective primary vectors, *Aedes aegypti* (Linnaeus, 1762) and *Anopheles darlingi* Root, 1926 (Culicidae) [7]. These species occupy distinct ecological niches, as *Ae. aegypti* is predominantly urban and highly adapted to human settlements, while *A. darlingi* thrives in rural and peri-urban areas, particularly near forest edges and river basins [5].

Despite these different habitats, both mosquitoes are managed through the intensive use of synthetic insecticides, notably pyrethroids which continuous chemical pressure has triggered the widespread emergence of resistant populations, as the prolonged exposure to these compounds selects for individuals with enhanced survival mechanisms [8].

In susceptible mosquito populations, the application of pyrethroids triggers a cascade of physiological events beyond immediate neurotoxicity, which the interaction induces an overproduction of reactive oxygen species, such as the superoxide radical (O_2_^•−^), hydrogen peroxide (H_2_O_2_), and the highly reactive hydroxyl radical (^•^OH) [9].

This redox imbalance leads to acute oxidative stress and potential cellular damage, forcing the larvae to recruit a complex antioxidant and defense system to mitigate the toxic challenge [10]. Within this framework, enzymes such as superoxide dismutase (SOD) and catalase (CAT) act as the primary enzymatic lines of defense to mitigate the toxic challenge and maintain cellular homeostasis [11].

Regarding this metabolic context, the efficiency of the response depends on the individual level of susceptibility, where the insecticide may act either as a substrate for enzymatic degradation or as a potent inhibitor of key metabolic pathways [12]. Enzymes such as glutathione S-transferases (GSTs), esterases, and cytochrome P450 monooxygenases (MFOs) are central to this detoxification process [13]. Their modulation determines whether the larvae can neutralize the chemical threat or succumb to systemic failure caused by the inability to process these exogenous molecules [14].

Furthermore, a primary site of action for many neurotoxic substances is the inhibition of acetylcholinesterase (AChE), an essential enzyme for terminating nerve impulses at the synaptic cleft [15]. The disruption of AChE activity, combined with the potential failure of the antioxidant and detoxification machinery, is often decisive for larval mortality [16].

Understanding these multi-target interactions is crucial for developing new biopesticides capable of overcoming the survival mechanisms evolved by *Ae. aegypti* and *An. darlingi* [17]. Given the limitations imposed by insecticide resistance, the search for sustainable alternatives has shifted towards botanical insecticides [18].

Essential oils represent a prominent class of these derivatives, being frequently extracted via hydrodistillation from various plant parts and subsequently characterized through advanced analytical techniques to identify their diverse secondary metabolites, which primarily include monoterpenes and sesquiterpenes, among other bioactive classes [19].

Notably, the insecticidal effects triggered by these essential oils often mirror the biochemical disruptions reported for pyrethroids, including the modulation of antioxidant defenses and the inhibition of vital enzymatic targets [20]. For instance, essential oils from *Piper betle* L. (Piperaceae) and *Sphaeranthus indicus* L. (Asteraceae) have demonstrated significant activity against *A. aegypti* (LC_50_ of 48.6 and 51.3 µg/mL) accompanied by a 1.5 to 2-fold increase in GST activity relative to the control, while cytochrome P450 activity was reduced by approximately 40–50% [21].

While these biochemical assessments provide evidence of metabolic interference, the specific binding modes and molecular interactions between botanical secondary metabolites and these enzymatic targets can be further elucidated through molecular docking simulations [22]. This computational approach allows for a precise investigation of the inhibitory mechanisms at the atomic level, identifying the affinity and stability of the complexes formed between bioactive molecules and key enzymes such as GST, AChE, and cytochrome P450 [23].

The effectiveness of essential oils is further supported by extensive screenings in the literature. A comprehensive study investigating 337 essential oils from families such as Lamiaceae, Lauraceae, Myrtaceae, and Piperaceae revealed that 60% possessed larvicidal action with LC_50_ values below 100 µg/mL. Furthermore, 17% of the tested exhibited high toxicity, with LC_50_ values lower than 10 µg/mL. Among these botanical groups, the Piperaceae family was identified as the most active, highlighting its potential as a primary source of bioactive molecules for vector control [24].

The family Piperaceae stands out in this context due to its remarkable chemical diversity and biological potential [25]. Specifically, the genus *Piper* comprises over 2000 species distributed across tropical and subtropical regions, being widely recognized for its production of bioactive amides, lignans, and terpenes [26].

Studies have already demonstrated the efficacy of essential oils from *Piper* against Culicidae larvae, as their complex chemical profiles act on multiple physiological sites simultaneously [27,28]. This multi-target action not only ensures high mortality rates but also reduces the likelihood of resistance development [29].

Among this vast diversity, *Piper humillimum* C.DC. is morphologically characterized by an erect growth habit, ovate to elliptical leaves with a short-acuminate apex, and a spike-type inflorescence featuring fringed floral bracts [30]. The leaf blades exhibit a smooth surface with conspicuous translucent glands and a distinct brochidodromous venation pattern, while the floral structures are marked by triangular to subpeltate bracts. Furthermore, this species is distinguished by a sessile fruit structure with three stigmas and an inconspicuous or absent style, being widely distributed across the northern states of Brazil, such as Amazonas [31].

Despite its formal taxonomic description and regional occurrence, there are currently no records in the scientific literature regarding the chemical composition of its essential oil or its biological properties. Therefore, this study aims to provide the first chemical characterization of essential oil from *P. humillimum* and to evaluate its larvicidal efficacy against *Ae. aegypti* and *An. darlingi.* By integrating *in vivo* bioassays and *in vitro* biochemical assessments with molecular docking simulations, this work seeks to reveal the biological potential of this Amazonian species and provide a comprehensive understanding of its multi-target action, contributing to the development of sustainable strategies for vector control.

## 2. Materials and Methods

### 2.1. Chemicals and Reagents

The reagents and standards used in this study included methanol reference standards, 2 M hydrochloric acid (HCl), 10% trichloroacetic acid (TCA), 0.5% (*w*/*v*) thiobarbituric acid (TBA), TBARS reagent, 10 M potassium iodide (KI), 250 mM hydrogen peroxide (H_2_O_2_), bovine serum albumin (BSA), malondialdehyde (MDA) standard solution, 10 mM 2,4-dinitrophenylhydrazine (DNPH), ethanol, ethyl acetate, 6 M guanidine, 50% formic acid, L-glutamic acid, glutamate oxidase, horseradish peroxidase, potassium phosphate, HEPES, sodium phosphate, EDTA, sodium acetate, N,N,N′,N′-tetramethylethylenediamine (TEMED), quercetin, and dimethylformamide.

Additional compounds comprised α-cypermethrin, nicotinamide adenine dinucleotide phosphate (NADPH), reduced glutathione (GSH), glutathione reductase (GR), acetylcholinesterase (AChE), acetylthiocholine iodide (ATCI), 5,5′-dithiobis-(2-nitrobenzoic acid) (DTNB), 1-chloro-2,4-dinitrobenzene (CDNB), ethacrynic acid, 3,3′,5,5′-tetramethylbenzidine (TMBZ), α-naphthyl acetate, β-naphthyl acetate, Fast Blue reagent, sodium dodecyl sulfate (SDS), eserine (physostigmine), neostigmine, dimethyl sulfoxide (DMSO), and 10% glucose solution were of analytical grade and were purchased from Merck (São Paulo, SP, Brazil).

Germacrene D (99%) and δ-cadinene (99%) were obtained from the Laboratory of Active Principles of the Amazon (LAPAAM), Amazonas, AM, Brazil.

### 2.2. Collection and Taxonomic Identification of P. humillimum

*P. humillimum* samples were collected at the National Institute for Amazonian Research (INPA), located in the city of Manaus, Amazonas, Brazil (3°05′45″ S, 59°59′23″ W). Taxonomic identification was performed by the botanist Gabriel Mendes Marcusso, and a voucher specimen was deposited in the INPA herbarium under accession number 44127.

Prior to essential oil extraction, the leaves were thoroughly washed with tap water, shade-dried at room temperature, ground into a fine powder, and stored in a sealed plastic container until further processing [32].

#### Extraction and Analysis of Essential Oil Composition by GC–MS and GC–FID

The extraction of essential oil from *P. humillimum* leaves (200 g) was conducted in accordance with the methodology previously described [32]. Prior to chromatographic injection, the essential oil (1 mg) was diluted in ethyl acetate (1 mL).

Gas chromatography–mass spectrometry (GC–MS) analyses were performed using a Trace GC Ultra model coupled to an ISQ single quadrupole mass spectrometer (both from Thermo Scientific, Waltham, MA, USA). For each sample, 1 µL was injected with a split ratio of 1:50 onto a capillary column TR-5 (30 m × 0.25 mm × 0.25 um). The oven temperature was programmed from 60 °C to 240 °C at a heating rate of 3 °C/min, utilizing helium as the carrier gas. The injection and interface temperatures were set at 240 °C and 250 °C, respectively. The mass spectrometer operating parameters were: ion source temperature of 220 °C, electron ionization voltage of 70 eV, mass range of m/z 40–600, and a scan speed of 2 s. Data acquisition and processing were controlled via Xcalibur software v.2.2. Volatile compounds were identified by comparing their experimental mass spectra with commercial libraries (NIST, Adams, and an in-house library).

Gas chromatography–flame ionization detection (GC–FID) analyses were performed on an Agilent G-1860 Plus system (Agilent Technologies, Santa Clara, CA, USA) equipped with an apolar HP-5 capillary column (30 m × 0.32 mm × 0.25 um). The oven temperature program was identical to that used for GC–MS, rising from 60 °C to 240 °C at 3 °C/min, using hydrogen as the carrier gas at a flow rate of 4 mL/min. The injector temperature was maintained at 250 °C with a split ratio of 1:2. The flame ionization detector was operated at 280 °C with hydrogen, synthetic air, and nitrogen flow rates maintained at 30, 300, and 30 mL/min, respectively. Automated injections of 1 µL were managed by an automatic liquid sampler under HP Chemstation software (version B.04.03, Agilent Technologies, Santa Clara, CA, USA) control. Retention indices (RI) were experimentally determined under linear temperature gradient conditions using an *n*-alkanes series (C_8_–C_30_, Supelco) and calculated via the Van den Dool and Kratz equation. Final compound identification was confirmed by cross-referencing the obtained RIs with literature data.

### 2.3. Collection, Identification, and Rearing of Mosquito Larvae

Larvae of *Ae. aegypti* used in this study were obtained from laboratory colonies maintained at the Laboratory of Biological Control and Biotechnology of Malaria and Dengue under controlled environmental conditions (26 ± 3 °C, 85% relative humidity, and a 12:12 h light–dark photoperiod). Egg-bearing cellulose substrates were submerged in containers containing 2 L of tap water to stimulate hatching. After eclosion, larvae were reared on a commercial fish diet until reaching the fourth larval instar. Pupae were then carefully separated and transferred to plastic containers containing 50 mL of tap water, which were placed inside entomological cages (30 × 30 × 30 cm) for adult emergence. Emerged adults were maintained on a 10% glucose solution, and females were subsequently blood-fed on a *Mesocricetus auratus* (Cricetidae) hamster, following established procedures [32].

In parallel, adult *An. darlingi* mosquitoes were collected in the field at the Ramal da Cachoeira do Castanho, located in the municipality of Iranduba, Amazonas, Brazil (3º17′06″ S, 60º11′09″ W). Field collections were conducted in accordance with the guidelines described in the World Health Organization Manual on Malaria Entomology for Entomology and Vector Control Technicians [33]. Collected specimens were transported to the laboratory and identified using standard morphological taxonomic keys [34]. Following identification, *An. darlingi* larvae were reared under the same controlled conditions described for *Ae. aegypti*, ensuring methodological consistency across species.

#### 2.3.1. Larvicidal Activity Assay

The larvicidal assay evaluating the activity of the essential oil, germacrene D, and δ-cadinene were conducted in accordance with the recommendations of the World Health Organization for laboratory testing of mosquito larvicides [35] under controlled conditions (28 ± 2 °C, relative humidity of 80 ± 5%). In brief, groups of 25 larvae (third instar) of *Ae. aegypti* (*n* = 250) and *An. darlingi* (*n* = 250) were separately distributed into 25 plastic containers (200 mL capacity), each containing 99 mL of distilled water and concentrations 20 to 100 µg/mL of essential oil, germacrene D, and δ-cadinene. These solutions were prepared using DMSO as a solute vehicle, ensuring that the final concentration of DMSO in the working solution remained well below 1% to avoid any subtoxic or confounding effects on the larvae, as validated by our negative control and subsequent biochemical assays.

α-Cypermethrin (0.21 μg/mL) [36] (de Oliveira et al., 2022) and DMSO (20 to 100 μg/mL) were used as positive and negative controls, respectively. The positive control was employed strictly to confirm colony susceptibility and was excluded from the downstream analysis of variance (ANOVA) to maintain statistical clarity and independence. All treatments were performed in quintuplicate (5 technical replicates per concentration, containing 25 larvae each). The entire experiment was repeated five times using different biological batches of larvae (5 independent biological replicates, totaling 125 larvae per concentration per species), with five independent replicates. The percentage of activity at each concentration was calculated after a 48 h exposure period using the equation: larvicidal activity (%) = (number of dead larvae/total number of larvae) × 100.

#### 2.3.2. Residual Effects Assay

To assess the residual larvicidal effects of the essential oil, germacrene D, and δ-cadinene over time, groups of *Ae. aegypti* (*n* = 100) and *An. darlingi* (*n* = 100) larvae were exposed to their respective LC_90_ concentrations, previously established in larvicidal bioassays. Specifically, *Ae. aegypti* and *An. darlingi* were treated with essential oil at 70.41 and 86.97 µg/mL, germacrene D at 82.18 and 79.03 µg/mL, and δ-cadinene at 79.15 and 71.93 µg/mL, respectively. For comparison, α-cypermethrin was included as a reference larvicide and tested at 0.21 µg/mL for both species.

For each treatment, the larvae were divided into 4 technical replicates of 25 larvae each, and the entire assay was repeated in triplicate with independent biological batches. The experiments were conducted in containers holding 200 mL of water, and larval mortality was monitored at 24 and 48 h intervals for seven consecutive days. At each assessment, dead larvae were removed and replaced with viable individuals to maintain constant larval density. Residual larvicidal efficacy was defined as sustained mortality equal to or exceeding 90% throughout the evaluation period [37] (de Oliveira et al., 2025).

### 2.4. Integrated Evaluation of Mechanisms of Action

#### 2.4.1. Preparation of the Homogenate

Homogenate preparation was performed immediately after the exposure period of the larvicidal [38] (de Oliveira et al., 2025). Larvae of *Ae. aegypti* (*n* = 50) and *An. darlingi* (*n* = 50) that died during exposure to the essential oil, germacrene D, and δ-cadinene (100 µg/mL) were harvested immediately upon death. To prevent any postmortem biochemical degradation and non-specific tissue oxidation, all specimens were instantly transferred into 10 mL tubes containing 2.5 mL of ice-cold potassium phosphate buffer (0.1 M, pH 7.3). This immediate cooling and thermal shock were strictly sustained throughout the entire harvesting and preparation procedure to arrest enzymatic activity at the exact moment of death. α-Cypermethrin (0.21 µg/mL) and DMSO (50 µg/mL) were included as positive and negative controls, respectively.

The entire homogenization process was performed using a vortex mixer for 5 min, followed by centrifugation at 4000 rpm for 5 min under refrigerated conditions (4 °C). The resulting supernatants were transferred to Eppendorf tubes and maintained at 4 °C until further analyses.

The obtained supernatants were used for both *ex vivo* enzymatic activity measurements and *in vitro* inhibition assays, depending on the experimental approach. In the *ex vivo* analyses, enzyme activities reflected the physiological effects resulting from specimens exposure to xenobiotics. In contrast, for *in vitro* assays, supernatants obtained from DMSO treated control were subsequently incubated with xenobiotics to assess their direct inhibitory effects on enzymatic activity.

Enzyme-specific absorbance was measured using a microplate reader at wavelengths optimized for each assay. All measurements were performed in technical triplicate, and mean absorbance values were calculated to ensure data reliability and reproducibility.

#### 2.4.2. Determination of Protein Concentration

Protein concentrations in the larvae supernatants were determined using bovine serum albumin as the reference standard [39].

#### 2.4.3. *Oxidative Stress and Cellular Damage Markers*

##### Measurement of H_2_O_2_ Levels

The generation of reactive oxygen species was assessed by determining H_2_O_2_ levels, employed as a representative ROS marker [40]. Briefly, 45 µL of each supernatant sample was combined with an equal volume (45 µL) of potassium iodide solution (10 M). Absorbance was recorded at 390 nm using a microplate spectrophotometer.

H_2_O_2_ concentrations were quantified by interpolation from a calibration curve prepared from a 250 mM H_2_O_2_ stock solution and serially diluted to obtain concentrations ranging from 0 to 45 µmol. Data were expressed as µmol H_2_O_2_ min^−1^ mg^−1^ protein, and all determinations were carried out in technical triplicate.

##### Measurement of Lipid Oxidative Damage

Lipid oxidative injury induced by H_2_O_2_ was evaluated by estimating MDA formation, used as a marker of membrane lipid peroxidation. The analysis was performed using the TBARS [41], and later adapted [9]. For this procedure, 125 µL of each homogenate sample was incubated with 250 µL of a TCA–TBA reagent solution containing 10% trichloroacetic acid and 0.5% (*w*/*v*) thiobarbituric acid.

Upon completion of the reaction, absorbance was measured at 535 nm using a microplate reader. To correct for nonspecific turbidity, absorbance values obtained at 600 nm were subtracted from the primary readings. Lipid peroxidation levels were expressed as nmol MDA mg^−1^ protein. All measurements were performed in technical triplicate to ensure analytical robustness and reproducibility.

##### Measurement of Protein Oxidative Damage

Protein oxidative damage was assessed by quantifying carbonyl groups formed through oxidative modification of amino acid side chains, a process primarily mediated by highly reactive hydroxyl radicals generated from ROS. Carbonyl formation was determined based on the derivatization of oxidized proteins with DNPH, yielding stable hydrazone derivatives suitable for spectrophotometric detection. For this assay, 100 µL of insects homogenate was incubated with 150 µL of DNPH solution (10 mM in 2 M hydrochloric acid) for 1 h under controlled conditions. Proteins were subsequently precipitated by the addition of 250 µL of 6% TCA, followed by centrifugation at 1000 rpm for 5 min.

The resulting pellets were washed three times with an ethanol–ethyl acetate mixture to remove excess reagent and potential interferents. Protein solubilization was then achieved using 1 mL of 6 M guanidine hydrochloride prepared in 50% formic acid. Absorbance was measured at 366 nm using a microplate reader Multiskan GO Microplate Spectrophotometer (Thermo Fisher Scientific, Waltham, MA, USA). Protein oxidation levels were expressed as nmol carbonyls mg^−1^ protein. All measurements were performed in technical triplicate to ensure analytical reliability [42].

### 2.5. Antioxidant Defense System

#### 2.5.1. Measurement of SOD Activity

SOD activity was evaluated using the method originally described by [43], and later adapted for insect supernatants [44]. In brief, each supernatants previously mixed with 250 µL of HEPES buffer (20 mM, pH 7.0) and centrifuged at 15,000 rpm for 10 min at 4 °C to obtain clarified extracts. The reaction medium consisted of sodium phosphate buffer (0.025 M, containing 0.1 mM EDTA, pH 10), TEMED, and 10 µL of the clarified supernatant, with the enzymatic reaction initiated by the addition of 0.15% quercetin dissolved in dimethylformamide.

Quercetin autoxidation was monitored at 406 nm for 2 min. SOD activity was calculated as the amount of protein required to inhibit 50% of quercetin oxidation and expressed as U mg^−1^ protein. All measurements were performed in technical triplicate.

#### 2.5.2. Measurement of CAT Activity

CAT activity was evaluated by measuring the rate of H_2_O_2_ decomposition following the kinetic approach proposed by Aebi [45], with slightly modifications [46]. The assay was conducted at 25 °C using a reaction medium composed of phosphate buffer (0.1 M, pH 7.3) supplemented with H_2_O_2_ (300 mM). For each determination, 20 µL of the supernatant was added to 1.8 mL of buffer containing 180 µL of H_2_O_2_, and the decline in absorbance was continuously recorded at 240 nm for 2 min at 10 s intervals using a UV–visible spectrophotometer (Evolution 201, Thermo Scientific, Waltham, MA, USA). Catalase activity was calculated based on the rate of H_2_O_2_ consumption and expressed as µmol H_2_O_2_ min^−1^ mg^−1^ protein. All procedures were performed in triplicate.

### 2.6. Xenobiotic Detoxification System

#### 2.6.1. Measurement of GST Activity

*In vivo*: GST activity was determined using CDNB as the conjugation substrate. For the reaction, 20 µL of each supernatant was added to a reaction medium containing EDTA (2.5 mM), reduced glutathione (GSH, 0.1 M), phosphate buffer (0.25 M, pH 7.0), and distilled water, forming a final volume of 270 µL (Solution A). The mixture was equilibrated at 25 °C, after which the reaction was initiated by the addition of 10 µL of CDNB solution (25 mM). The increase in absorbance was recorded at 340 nm at 10 s intervals over a 5 min period using a microplate reader. GST activity was calculated from the rate of conjugate formation and expressed as µmol min^−1^ mg^−1^ protein. All assays were performed in technical triplicate to ensure analytical reliability [47].

*In vitro*: To assess the direct effects on GST activity, 20 µL aliquots of supernatants obtained from specimens treated exclusively with DMSO were pre-incubated with essential oil, germacrene D, or δ-cadinene (100 µg/mL), as well as with α-cypermethrin (0.21 µg/mL), for 20 min at 25 °C, with solvent concentrations maintained constant across all treatments and controls.

GST activity was subsequently determined using a colorimetric assay based on the conjugation of GSH with CDNB. Absorbance was measured at 340 nm using a microplate reader, and enzymatic activity was expressed relative to the corresponding DMSO control. Solvent-only incubations were included in all assays, ethacrynic acid (50 µg/mL) was used as a positive control, and reactions lacking supernatant were performed to exclude optical or chemical interference. All assays were conducted in technical triplicate.

#### 2.6.2. Measurement of MFO Activity

*In vivo:* For MFO activity, a sodium acetate buffer was prepared by diluting sodium acetate in distilled water and adjusting the pH to 5.0. The chromogenic reagent was obtained by dissolving TMBZ in methanol and subsequently diluting it in sodium acetate buffer (0.25 M, pH 5). Then, 200 µL of the TMBZ working solution, 25 µL of H_2_O_2_ (3%), and 20 µL of each sample supernatant were dispensed into individual wells of a microplate.

Plates were incubated at room temperature for 10 min to allow color development, after which absorbance was recorded at 620 nm using a microplate reader. Enzyme activity was expressed as nmol cyt c min^−1^ mg^−1^ protein, presented as mean values with mean ± standard error. All assays were performed in technical triplicate [48].

*In vitro:* Aliquots of supernatants obtained from specimens treated with DMSO were pre-incubated for 20 min at 25 °C with essential oil, germacrene D, or δ-cadinene (10 µg/mL), as well as with α-cypermethrin (0.21 µg/mL), with solvent concentrations maintained constant across all treatments and controls.

Following pre-incubation, MFO activity was determined using a colorimetric assay based on the oxidation of TMBZ in the presence of H_2_O_2_, prepared in sodium acetate buffer (0.25 M, pH 5.0). The reaction was initiated by the sequential addition of the chromogenic reagent and H_2_O_2_, and absorbance was measured at 620 nm using a microplate reader.

Enzymatic activity was expressed relative to the corresponding DMSO control. Solvent-only incubations were included in all assays, while reactions lacking supernatant were performed to exclude non-enzymatic or optical interference. All assays were conducted in technical triplicate [48].

#### 2.6.3. Measurement of α- and β-Esterase Activity

*In vivo:* Esterase activity was assessed using α-naphthyl acetate and β-naphthyl acetate as substrates in a kinetic colorimetric assay. Briefly, 10 µL of each supernatant was combined with 200 µL of substrate solution (0.3 mM) prepared in phosphate buffer and incubated at room temperature for 15 min. The enzymatic reaction was terminated by the addition of 50 µL of Fast Blue reagent, freshly prepared in Milli-Q (Millipore, Billerica, MA, USA) water containing sodium dodecyl sulfate (SDS).

Following an additional incubation period of 5 min to allow chromophore formation, absorbance was measured at 570 nm. Esterase activity was calculated based on the formation of α-naphthol and β-naphthol and expressed as µmol min^−1^ mg^−1^ protein, with results reported as mean ± standard error. All assays were performed in technical triplicate [48].

*In vitro:* To assess the direct effects on esterase activity, supernatants obtained from specimens treated with DMSO were used as the basal enzymatic source. Aliquots of these supernatants were pre-incubated for 20 min at 25 °C with essential oil, germacrene D, or δ-cadinene (100 µg/mL), as well as with α-cypermethrin (0.21 µg/mL), all prepared in DMSO, with the final solvent concentration kept constant across all treatments and controls.

The enzymatic reaction was initiated by the addition of α-naphthyl acetate or β-naphthyl acetate and conducted under the same conditions described for the in vivo assays. Reactions were terminated by the addition of Fast Blue reagent, and absorbance was measured at 570 nm using a microplate reader.

Esterase activity was expressed relative to the corresponding DMSO control. Solvent-only incubations were included in all assays, while eserine (physostigmine, 50 µg/mL) was used as a positive control for esterase inhibition. Additional reactions containing xenobiotics in the absence of supernatant were performed to exclude optical or chemical interference. All assays were conducted in technical triplicate [48].

#### 2.6.4. *Neurotoxicity Marker*

##### Measurement of AChE Activity

*In vivo*: The AChE was assessed using a colorimetric microplate assay based on the hydrolysis of acetylthiocholine and subsequent reaction with DTNB. The reaction system was assembled by mixing distilled water, phosphate buffer (100 mM, pH 7.3), DTNB solution (10 mM), AChI (8 mM), and 20 µL of sample supernatant in each well. The enzymatic reaction was carried out at 25 °C under light-protected conditions, and absorbance was recorded at 412 nm at 30 s intervals over a 2 min period using a microplate reader. AChE activity was calculated from the rate of chromophore formation and expressed as μmol/min/mg of protein. All assays were performed in technical triplicate to ensure analytical reliability [37].

*In vitro:* AChE activity was evaluated under cell-free conditions using supernatants obtained from specimens treated exclusively with DMSO. Aliquots of these supernatants were pre-incubated for 20 min at 25 °C with essential oil, germacrene D, or δ-cadinene (100 µg/mL), as well as with α-cypermethrin (0.21 µg/mL), all prepared in DMSO.

AChE activity was subsequently determined using a colorimetric microplate assay based on the hydrolysis of acetylthiocholine and its reaction with DTNB, following the method described [49], with minor adaptations. Absorbance was monitored at 412 nm for 2 min at 25 °C, and enzymatic activity was expressed relative to the corresponding DMSO control. Solvent-only incubations were included in all assays, neostigmine (5 µg/mL) was used as a positive control for AChE inhibition, and all assays were performed in technical triplicate.

### 2.7. Molecular Docking Studies

Computational analyses were conducted to investigate the inhibitory potential of germacrene D, δ-cadinene, and α-cypermethrin against GST and AChE. The 3D structures of germacrene D (CID 5317570), α-cypermethrin (CID 10223), and δ-cadinene (CID 93357) were retrieved from the PubChem database.

To ensure chemical accuracy, ligands were refined by evaluating their ionization states and most stable tautomeric forms at physiological pH (7.4) using MarvinSketch version 23.1 (Chemaxon, Budapest, Hungary). Subsequently, geometry optimization was performed via the PM7 semi-empirical method in MOPAC2016 to identify minimum-energy conformations [50].

The crystallographic targets were obtained from the RCSB Protein Data Bank: AChE from *A. gambiae* (PDB ID: 6ARY; 2.26 Angstrom (Å) resolution), carrying the G119S mutation, a substitution known to compromise the efficacy of several insecticide classes [51] and GST from *A. gambiae* (PDB ID: 1PN9; 2.00 Å resolution), carrying the agGSTd1-6 mutations, associated with DDT metabolism and pyrethroid inhibition [52].

Prior to docking, all non-essential molecules were removed from the protein structures. Docking simulations were performed using AutoDock Vina version 1.2.0 (The Scripps Research Institute, La Jolla, CA, USA), with the search space defined to encompass the entire catalytic gorge of both enzymes. Detailed configuration parameters, including grid box centers, dimensions, energy range, exhaustiveness, and spacing (0.375 Å), are provided in Supplementary Material Table S1.

To validate the protocol, the co-crystallized ligands 2-acetamido-2-deoxy-β-D-glucopyranose (NAG) and S-hexylglutathione (GTX) were re-docked under identical conditions, with the resulting root-mean-square deviation (RMSD) values confirming the accuracy of the setup. Resulting ligand–enzyme complexes were visualized using Discovery Studio Visualizer version 21.1.0 (Dassault Systèmes, Vélizy-Villacoublay, France) to identify key molecular interactions.

Complementary kinetic assays were conducted to elucidate the mode of enzyme inhibition. The inhibition pattern and steady-state inhibition constant (Ki) were determined through analysis of Lineweaver–Burk double-reciprocal plots, providing experimental support for the interaction mechanisms suggested by the *in silico* results [37].

### 2.8. Statistical Analyses

Descriptive statistics were used to determine the mean and standard deviation (SD) for all variables. Data normality and homogeneity of variances were verified for all datasets using the Shapiro–Wilk and Brown–Forsythe tests, respectively. Larvicidal activity data were analyzed using log-probit dose–response regression to estimate LC_50_ and LC_90_ values with 95% confidence intervals, slope, and χ^2^ goodness-of-fit parameters. Comparisons among LC values were performed by one-way ANOVA applied to log-transformed estimates obtained independently for each biological replicate to ensure statistical variance.

The residual larvicidal effect and all biochemical and enzymatic data (in vivo and in vitro) were analyzed by one-way ANOVA followed by Tukey’s post hoc test. All statistical analyses were performed with a significance level of *p* < 0.05. Regression analyses and graphs were generated using GraphPad Prism version 9.3, while all other statistical procedures were conducted using IBM SPSS Statistics version 21 (IBM Corp., Armonk, NY, USA) [53].

## 3. Results

### 3.1. Extraction and Analysis of Essential Oil Composition by GC–MS and GC–FID

The hydrodistillation of *P. humillimum* leaves yielded a significant essential oil content of 3.5 ± 0.4% (*v*/*w*). GC-MS and GG-FID analysis led to the identification of eight main constituents, accounting for 100% of the total oil composition (Table 1). The chemical profile was overwhelmingly dominated by sesquiterpene hydrocarbons, which represented 91.47% of the total integrated area. Germacrene D (61.51%) was the primary substance, followed by δ-cadinene (17.46%) and β-cubebene (8.36%).

Other sesquiterpenes, such as germacrene B (2.04%), γ-elemene (1.81%), β-caryophyllene (1.07%), and γ-muurolene (1.04%), were detected in lower proportions. Aliphatic ester was represented solely by isobutyl acetate, which accounted for 6.71% of the overall profile.

### 3.2. Larvicidal Activity Assay

**Figure 1:**
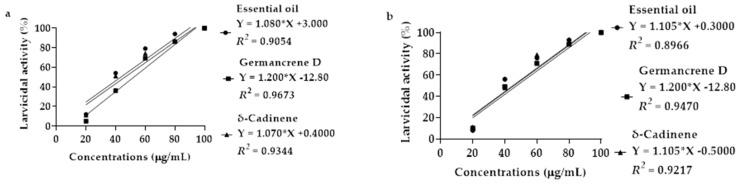
Mortality of *Ae. aegypti* (**a**) and *An. darlingi* (**b**) larvae following 48 h of exposure to essential oil, germacrene D, and δ-cadinene. α-Cypermethrin at 0.21 μg/mL caused 100% of mortality. No mortality of larvae was registered in negative control DMSO at concentrations ranging from 20 to 100 μg/mL.

The larvicidal efficacy of *P. humillimum* essential oil and its major sesquiterpenes, germacrene D and δ-cadinene demonstrating a significant dose-dependent mortality against both *Ae. aegypti* and *An. darlingi* (Table 2, Figure 1a,b). Dose-dependent effect was confirmed for all samples, as evidenced by statistically significant slopes (*p* < 0.05) and high coefficients of determination (R^2^ > 0.89), which validate the reliability of the regression models.

In tests involving *Ae. aegypti*, the essential oil induced mortality rates ranging from 12% to 100%, exhibiting an LC_50_ of 37.42 µg/mL. For the isolated compounds, δ-cadinene caused mortality between 11% and 100%, resulting in an LC_50_ of 39.88 µg/mL, while germacrene D showed mortality from 5% to 100% with a comparatively higher LC_50_ of 46.04 µg/mL. Statistical analysis indicated that only essential oil was significantly more potent than germacrene D against this species (*p* < 0.0001).

Regarding the activity against *An. darlingi*, the essential oil resulted in mortality percentages from 8% to 100% with an LC_50_ of 42.50 µg/mL. Germacrene D showed a similar toxicological profile, with mortality between 10% and 100% and an LC_50_ of 42.25 µg/mL. Although δ-cadinene displayed the lowest numerical LC_50_ value of 34.75 µg/mL, no statistically significant differences were observed among the three samples for this vector according to Tukey’s post hoc test (*p* > 0.05). Throughout the experiments, the negative control (DMSO) registered no larval deaths, while the positive control α-cypermethrin at 0.21 µg/mL consistently caused 100% mortality.

Both conventional light and confocal microscopy images of the vector larvae are presented in Supplementary Material Figures S2 and S3.

#### Residual Effects Assay

**Figure 2:**
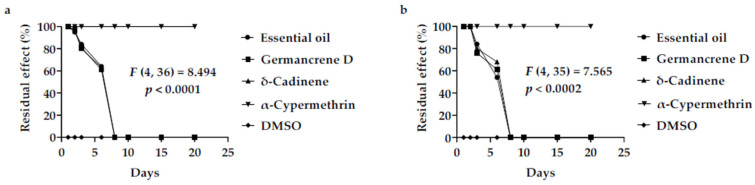
Residual effect of essential oil (70.41 and 86.97 μg/mL), germacrene D (82.18 and 79.03 μg/mL), δ-cadinene (79.15 and 71.93 μg/mL) over a 25-day monitoring period, comparison to α-cypermethrin (0.21 for both species), against *Ae. aegypti* (**a**) and *An. darlingi* (**b**) larvae. No larval mortality was observed in the negative control group treated with DMSO. Data are presented as mean ± standard deviation.

The residual larvicidal activity of the *P. humillimum* essential oil and its major substances was monitored over a 20-day period (Figure 2a,b). For both *Ae. aegypti* and *An. darlingi*, the substances maintained high efficacy, exceeding 75% mortality, only during the first 72 h of exposure. A significant reduction in biological activity was observed starting from the sixth day, with all treatments reaching 0% mortality by day 8.

In contrast, the positive control α-cypermethrin sustained 100% mortality throughout the entire 20-day monitoring period for both mosquito species. Throughout the evaluation, no larval deaths were recorded in the negative control group treated with DMSO. Statistical analysis confirmed significant differences between the treatment groups for both *Ae. aegypti* (F (4, 36) = 8.494, *p* < 0.0001) and *An. darlingi* (F (4, 35) = 7.565, *p* = 0.0002), highlighting the distinct mortality patterns recorded between the synthetic pyrethroid and the natural substances.

### 3.3. Oxidative Stress and Cellular Damage Markers

#### 3.3.1. Measurement of H_2_O_2_ Levels

Botanical treatments triggered a rapid and substantial accumulation of H_2_O_2_ in *Ae. aegypti* (*F* (4, 10) = 92.75; *p* < 0.0001) and *An. darlingi* (*F* (4, 10) = 208.8; *p* < 0.0001) (Table 3). In *Ae. aegypti*, the essential oil (61.00 ± 5.03 μmol H_2_O_2_ min^−1^ mg^−1^ protein) and its constituent δ-cadinene (55.67 ± 6.11 μmol H_2_O_2_ min^−1^ mg^−1^ protein) exhibited similar effect (*p* > 0.05), significantly outperforming germacrene D (36.67 ± 1.52 μmol H_2_O_2_ min^−1^ mg^−1^ protein) (*p* < 0.05).

Regarding *An. darlingi*, the induction profile was even more pronounced. Notably, the essential oil (74.00 ± 7.00 μmol H_2_O_2_ min^−1^ mg^−1^ protein) showed no statistical difference from α-cypermethrin (81.33 ± 1.52 μmol H_2_O_2_ min^−1^ mg^−1^ protein), highlighting its high efficacy as an oxidative stress inducer. In both species, all treatments represented a drastic increase over the basal activity of the DMSO control group (5.66 ± 2.51 and 4.00 ± 1.05 μmol H_2_O_2_ min^−1^ mg^−1^ protein, respectively).

#### 3.3.2. Measurement of Lipid Oxidative Damage

As a direct consequence of the initial H_2_O_2_ accumulation, lipid oxidative damage, measured by MDA levels, was significantly induced in *Ae. aegypti* (*F* (4, 10) = 113.9; *p* < 0.0001) and *An. darlingi* (*F* (4, 10) = 124.6; *p* < 0.0001). For *Ae. aegypti*, the essential oil (54.00 ± 5.00 nmol MDA mg^−1^ protein), germacrene D (43.3 ± 6.02 nmol MDA mg^−1^ protein), and δ-cadinene (47.35 ± 4.04 nmol MDA mg^−1^ protein) caused substantial lipid peroxidation with no statistical difference between these three treatments (*p* > 0.05).

In *An. darlingi* larvae, although the synthetic control showed the highest damage (81.67 ± 3.05 nmol MDA mg^−1^ protein), δ-cadinene (54.00 ± 4.35 nmol MDA mg^−1^ protein) was the most active among the natural treatments (*p* < 0.05). In both species, these results represented a massive increase compared to the DMSO control (1.33 ± 0.33 and 1.15 ± 0.20 nmol MDA mg^−1^ protein, respectively) (Table 3).

#### 3.3.3. Measurement of Protein Oxidative Damage

Driven by the sustained pro-oxidant state, protein oxidative damage, quantified through carbonyl content, was markedly elevated in *Ae. aegypti* (*F* (4, 10) = 193.4; *p* < 0.0001) and *An. darlingi* (*F* (4, 10) = 293.9; *p* < 0.0001). In *Ae. aegypti*, the highest protein damage was recorded for δ-cadinene (83.00 ± 2.64 nmol carbonyls mg^−1^ protein) and the essential oil (74.67 ± 6.02 nmol carbonyls mg^−1^ protein), which did not differ statistically from each other, including α-cypermethrin (77.33 ± 3.51 nmol carbonyls mg^−1^ protein).

For *An. darlingi*, the essential oil and isolated substances also caused severe protein carbonylation, with δ-cadinene (68.00 ± 3.00 nmol carbonyls mg^−1^ protein) showing a superior effect compared to germacrene D (58.33 ± 2.30 nmol carbonyls mg^−1^ protein) (*p* < 0.05). Basal levels for the DMSO control were 4.15 ± 1.05 and 3.90 ± 0.85 nmol carbonyls mg^−1^ protein, respectively (Table 3).

### 3.4. Antioxidant Defense System

#### 3.4.1. Measurement SOD Activity

In response to this mounting oxidative injury, the antioxidant defense was activated, with SOD activity significantly induced in both *Ae. aegypti* (*F* (4, 10) = 102.9; *p* < 0.0001) and *An. darlingi* (*F* (4, 10) = 661.7; *p* < 0.0001). For *Ae. aegypti*, the essential oil (87.33 ± 11.59 U mg^−1^ protein), germacrene D (83.31 ± 6.80 U mg^−1^ protein), and δ-cadinene (83.41 ± 2.01 U mg^−1^ protein) showed no statistical difference compared to the positive control α-cypermethrin (92.41 ± 3.21 U mg^−1^ protein) (*p* > 0.05).

In *An. darlingi*, α-cypermethrin reached the highest SOD induction (104.00 ± 1.06 U mg^−1^ protein), followed by δ-cadinene (95.00 ± 3.60 U mg^−1^ protein). The DMSO control groups exhibited activities of 12.33 ± 2.15 and 10.50 ± 1.80 U mg^−1^ protein, respectively (Table 4).

#### 3.4.2. Measurement CAT Activity

To specifically counteract the primary accumulation of H_2_O_2_, CAT activity showed significant elevation in *Ae. aegypti* (*F* (4, 10) = 166.0; *p* < 0.0001) and *An. darlingi* (*F* (4, 10) = 599.9; *p* < 0.0001) (Table 4). In *Ae. aegypti*, no statistical variance was found between the essential oil (74.31 ± 7.02 μmol H_2_O_2_ min^−1^ mg^−1^ protein), germacrene D (79.00 ± 9.16 μmol H_2_O_2_ min^−1^ mg^−1^ protein), and δ-cadinene (89.61 ± 2.01 μmol H_2_O_2_ min^−1^ mg^−1^ protein) (*p* > 0.05).

For *An. darlingi*, the essential oil (82.09 ± 1.03 μmol H_2_O_2_ min^−1^ mg^−1^ protein), germacrene D (76.33 ± 4.50 μmol H_2_O_2_ min^−1^ mg^−1^ protein) and δ-cadinene (79.67 ± 3.21 μmol H_2_O_2_ min^−1^ mg^−1^ protein) represented a massive increase over the DMSO group (0.66 ± 0.01 and 0.58 ± 0.05 μmol H_2_O_2_ min^−1^ mg^−1^ protein, respectively).

### 3.5. Xenobiotic Detoxification System

#### 3.5.1. Measurement of GST Activity

The overwhelming oxidative stress further compromised the xenobiotic detoxification system, leading to a significant inhibition of *in vivo* GST activity in *Ae. aegypti* (*F* (4, 10) = 167.0; *p* < 0.0001) and *An. darlingi* (*F* (4, 10) = 316.8; *p* < 0.0001) (Table 5). In *Ae. aegypti*, the essential oil (11.01 ± 2.00 µmol min^−1^ mg^−1^ protein), germacrene D (15.67 ± 1.52 µmol min^−1^ mg^−1^ protein), and δ-cadinene (11.67 ± 1.52 µmol min^−1^ mg^−1^ protein) caused a severe reduction compared to the DMSO control (67.61 ± 5.89 µmol min^−1^ mg^−1^ protein).

In *An. darlingi*, the essential oil (15.31 ± 3.19 µmol min^−1^ mg^−1^ protein), germacrene D (18.67 ± 2.08 µmol min^−1^ mg^−1^ protein) and δ-cadinene (16.33 ± 1.52 µmol min^−1^ mg^−1^ protein) also inhibited the enzyme significantly (*p* < 0.05) compared to the basal activity (71.33 ± 4.50 µmol min^−1^ mg^−1^ protein). These findings were corroborated by *in vitro* assay (Supplementary Material Figure S3).

#### 3.5.2. Measurement MFO Activity

Concurrently, the monooxygenase system was recruited as a metabolic bypass, with MFO activity significantly induced across all treatments in *Ae. aegypti* (*F* (4, 10) = 59.53; *p* < 0.0001) and *An. darlingi* (*F* (4, 10) = 103.2; *p* < 0.0001) (Table 5). In *Ae. aegypti*, the essential oil (63.67 ± 6.42 nmol cyt c min^−1^ mg^−1^ protein), germacrene D (59.61 ± 5.03 nmol cyt c min^−1^ mg^−1^ protein) and δ-cadinene (61.17 ± 11.37 nmol cyt c min^−1^ mg^−1^ protein) were statistically comparable to α-cypermethrin (73.67 ± 6.42 nmol cyt c min^−1^ mg^−1^ protein) (*p* > 0.05).

In *An. darlingi*, the essential oil (59.33 ± 7.50 nmol cyt c min^−1^ mg^−1^ protein), germacrene D (70.21 ± 3.21), δ-cadinene (73.52 ± 6.42 nmol cyt c min^−1^ mg^−1^ protein) and α-cypermethrin (71.61 ± 3.51 nmol cyt c min^−1^ mg^−1^ protein) also formed a single statistical group (*p* > 0.05). These inductions represented a massive increase compared to the DMSO control (3.33 ± 1.16 and 5.33 ± 2.08 nmol cyt c min^−1^ mg^−1^ protein, respectively). Patterns of induction were further validated by *in vitro* assays (Supplementary Material Figure S4).

#### 3.5.3. Measurement α- and β-Esterase Activity

To aid in the biotransformation of the botanical substances, *α*-esterase activity was significantly induced in *Ae. aegypti* (*F* (4, 10) = 32.64; *p* < 0.0001) and *An. darlingi* (*F* (4, 10) = 59.81; *p* < 0.0001) (Table 5). In *Ae. aegypti*, the essential oil (38.41 ± 4.04 µmol min^−1^ mg^−1^ protein), germacrene D (56.61 ± 10.79 µmol min^−1^ mg^−1^ protein), and δ-cadinene (43.67 ± 12.86 µmol min^−1^ mg^−1^ protein) promoted a substantial increase over the DMSO control (*p* < 0.05).

In *An. darlingi*, the essential oil (42.67 ± 1.52 µmol min^−1^ mg^−1^ protein), germacrene D (61.31 ± 9.29 µmol min^−1^ mg^−1^ protein) and δ-cadinene (59.67 ± 3.05 µmol min^−1^ mg^−1^ protein) acted as potent stimulators.

Regarding β-esterases (Table 6), *Ae. aegypti* exhibited a gradient (*F* (4, 10) = 412.4; *p* < 0.0001) involving the essential oil (42.67 ± 3.05 µmol min^−1^ mg^−1^ protein), germacrene D (51.09 ± 1.52 µmol min^−1^ mg^−1^ protein) and δ-cadinene (61.47 ± 3.05 µmol min^−1^ mg^−1^ protein).

In *An. darlingi*, δ-cadinene triggered a significantly higher response (44.00 ± 2.64 µmol min^−1^ mg^−1^ protein) than germacrene D (31.67 ± 2.51 µmol min^−1^ mg^−1^ protein), and the essential oil (35.33 ± 3.05 µmol min^−1^ mg^−1^ protein) (*F* (4, 10) = 295.0). These *in vivo* patterns were further validated *in vitro* (Supplementary Materials Figures S5 and S6).

### 3.6. Neurotoxicity Marker

#### Measurement AChE Activity

Ultimately, this systemic metabolic collapse was coupled with severe neurotoxicity, as AChE activity was significantly reduced in *Ae. aegypti* (*F* (4, 10) = 252.5; *p* < 0.0001) and *An. darlingi* (*F* (4, 10) = 576.4; *p* < 0.0001) (Table 6). In *Ae. aegypti*, a uniform inhibitory pattern was observed between the essential oil (14.67 ± 1.52 μmol min^−1^ mg^−1^ protein), germacrene D (14.33 ± 3.78 μmol min^−1^ mg^−1^ protein) and δ-cadinene (15.00 ± 1.00 μmol min^−1^ mg^−1^ protein) (*p* > 0.05).

In *An. darlingi*, the essential oil (17.00 ± 1.00 μmol min^−1^ mg^−1^ protein), germacrene D (18.31 ± 2.51 μmol min^−1^ mg^−1^ protein) and δ-cadinene (18.00 ± 1.00 μmol min^−1^ mg^−1^ protein) also formed a single statistical group (*p* > 0.05). All botanical treatments caused a massive drop relative to the DMSO control (82.45 ± 5.30 and 78.90 ± 4.15 μmol min^−1^ mg^−1^ protein, respectively). These effects were validated by *in vitro* assays (Supplementary Materials Figure S7).

### 3.7. Molecular Docking Studies

The reliability of the molecular docking protocol was rigorously validated by redocking the co-crystallized ligands into their respective enzyme active sites. The Root-Mean-Square Deviation (RMSD) for the reference ligand NAG (AChE) was 0.6454 Angstrom, while for GTX (GST) it was 1.7153 Å. Both values are significantly below the 2.0 Å threshold, confirming that the computational parameters accurately replicated the experimental binding modes and ensuring the predictive precision for the botanical compounds investigated.

Molecular docking simulations elucidated the structural basis for the potent neurotoxicity observed *in vivo and in vitro* (Table 7). Both δ-cadinene and germacrene D demonstrated superior binding affinities for the AChE pocket compared to the reference ligands. Germacrene D and δ-cadinene yielded the most stable complexes, with binding energies of −7.9 and −7.8 kcal/mol, along with Ki µM = 1.63 and 1.95, respectively, outperforming NAG (−5.6 kcal/mol, 78.0 Ki µM) and α-cypermethrin (−6.8 kcal/mol, 10.40 Ki µM).

**Figure 3:**
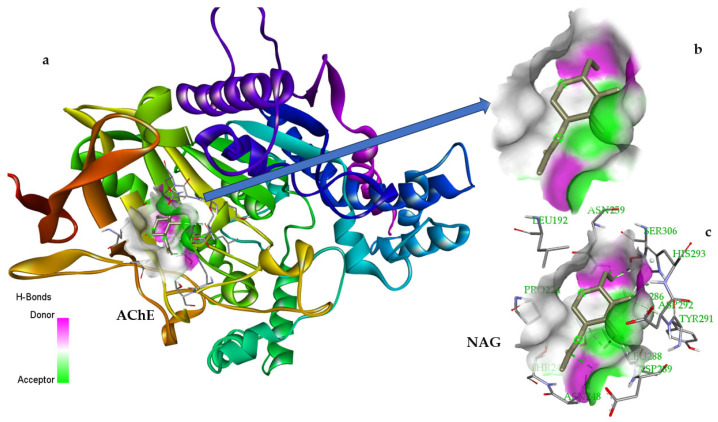
(**a**) General view of the enzyme AChE in ribbon-style (N-terminal to C-terminal colour gradient), highlighting the hydrogen-bonding surface mapping at the active site (scale: pink = donor; green = acceptor). The blue arrow indicates the transition to the magnified binding site analysis. (**b**) Upper inset highlighting the steric fit; the NAG molecule (beige sticks) is perfectly accommodated within the catalytic gorge, demonstrating high structural complementarity. (**c**) Lower inset detailing the specific interaction profile. The complex is stabilised by a prominent network of conventional hy-drogen bonds with Asn259, Ser306, Leu288, Asn248, and His293, with the latter also contributing pi-donor hydrogen bonds. Additionally, carbon-hydrogen bonds are established with Thr287, Asp292, and Asp289. The binding affinity is further enhanced by van der Waals interactions with Ala286, Tyr291, Leu192, Pro224, and Thr249.

The reference ligand NAG (Figure 3) was nestled within a solid potential surface, stabilized by a dense network of conventional hydrogen bonds with residues Asn259, Ser306, Leu288, Asn248, His293, and Thr287, demonstrating high steric and electrostatic complementarity. Surface mapping revealed that the sesquiterpenes are also perfectly accommodated within the hydrophobic domain of the catalytic gorge.

**Figure 4:**
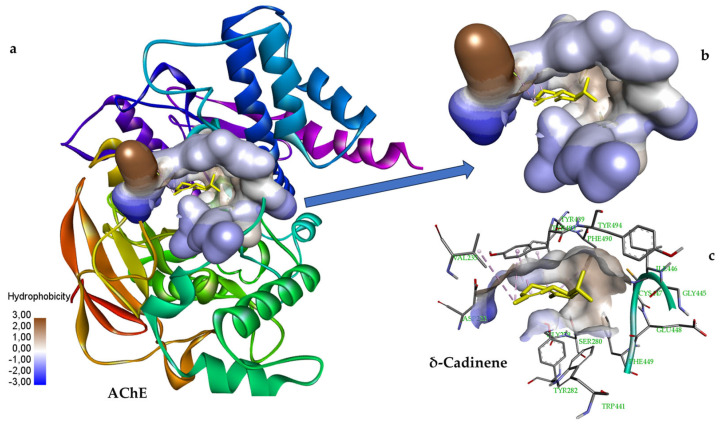
(**a**) General view of the enzyme AChE in ribbon-style (N-terminal to C-terminal colour gradient), highlighting the localized surface mapping at the active site. The blue arrow indicates the transition to the magnified binding site analysis. (**b**) Upper inset highlighting the steric fit; the δ-cadinene molecule is perfectly accommodated within the catalytic gorge, demonstrating high structural complementarity. (**c**) Lower inset detailing the specific interaction profile. The complex is stabilised by a prominent network of hydrophobic interactions, including an alkyl bond with Val235 and pi-alkyl interactions with Tyr493, Phe490, and Tyr489. The binding affinity is further enhanced by an extensive network of van der Waals interactions with Asp233, Trp441, Tyr494, Tyr282, Ser280, and Phe449.

**Figure 5:**
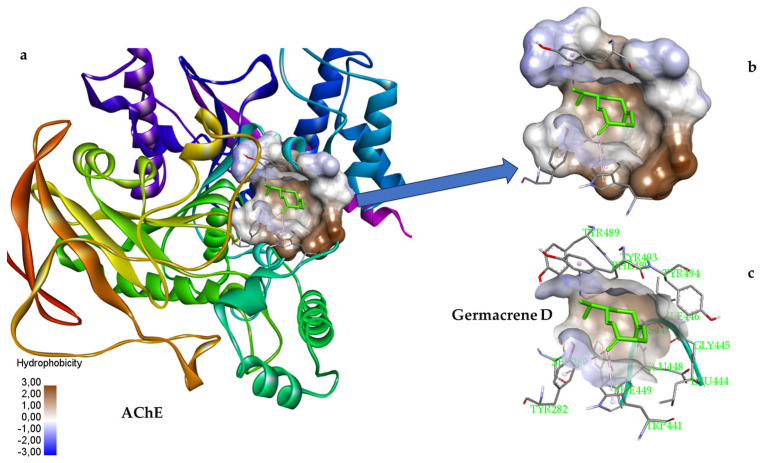
(**a**) General view of the enzyme AChE in ribbon-style (N-terminal to C-terminal color gradient), highlighting the localized hydrophobicity surface mapping at the active site (scale: brown = hydrophobic; blue = hydrophilic). The arrow indicates the transition to the magnified binding site analysis. (**b**) Upper inset highlighting the steric fit; the germacrene D molecule (green sticks) is perfectly accommodated within the hydrophobic domain of the catalytic gorge, demonstrating superior binding affinity. (**c**) Lower inset detailing the interaction profile; the residual transparent surface fragment reveals unique stabilization through pi-alkyl interactions with Tyr282 and Trp441, and a significant pi-sigma interaction with Tyr493. The complex is further stabilized by an extensive network of van der Waals interactions (Tyr494, Glu448, Cys447, Gly445, Ile446, Phe449, Phe490, and Tyr489), confirming high chemical complementarity.

**Figure 6:**
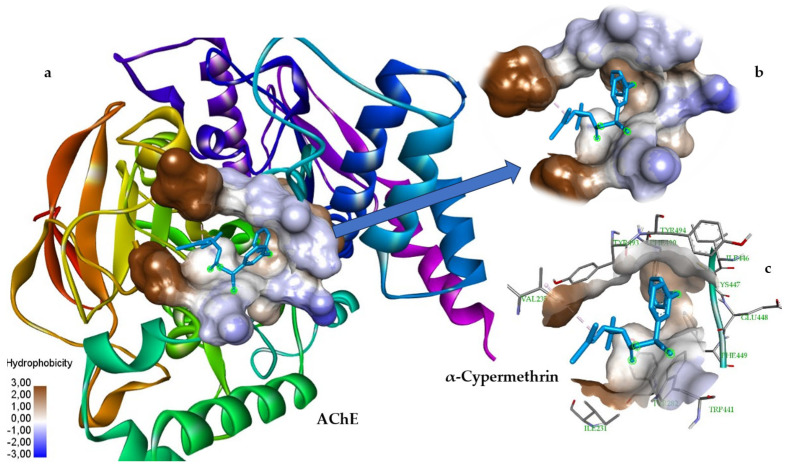
(**a**) General view of the enzyme (AChE) in ribbon-style (N-terminal to C-terminal color gradient), highlighting the localized hydrophobicity surface mapping at the active site (scale: brown = hydrophobic; blue = hydrophilic). The arrow indicates the transition to the magnified binding site analysis. (**b**) Upper inset highlighting the steric fit; the α-cypermethrin molecule (cyan sticks) is shown nestled within a solid hydrophobicity surface, demonstrating its high-affinity anchoring and efficient pocket filling. (**c**) Lower inset detailing the interaction profile; the residual transparent hydrophobic surface fragment reveals a stabilization profile dominated by a dense network of van der Waals interactions with key residues (Phe490, Phe449, Cys447, Ile446, Tyr493, Tyr282, Tyr494, Trp441, and Ile231), complemented by a specific alkyl bond with Val235. Dashed lines illustrate the binding interactions.

δ-Cadinene was anchored by a robust network of pi-alkyl interactions with Tyr493, Phe490, and Tyr489, along with a specific alkyl bond with Val235 (Figure 4). Germacrene D exhibited unique stabilization through pi-alkyl interactions with Tyr282 and Trp441, and a significant pi-sigma interaction with Tyr493 (Figure 5). In comparison, α-cypermethrin (Figure 6) was stabilized by a dense network of van der Waals interactions with key residues such as Phe490, Tyr494, and Trp441.

**Figure 7:**
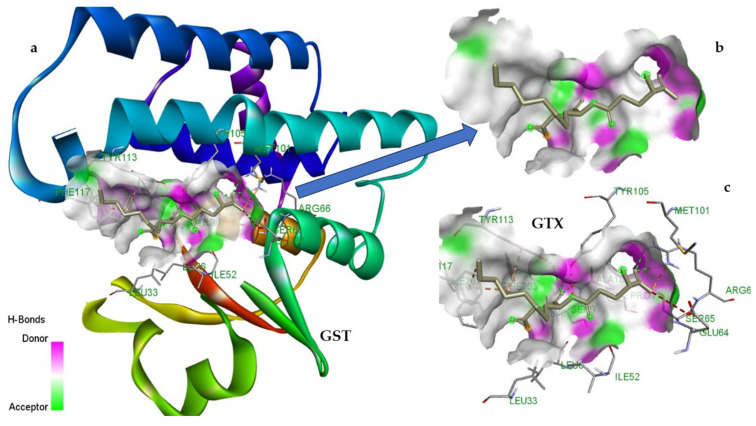
(**a**) General view of the enzyme (GST) in ribbon-style (N-terminal to C-terminal color gradient), highlighting the localized hydrogen-bond potential mapping at the active site. The arrow indicates the transition to the magnified binding site analysis. (**b**) Upper inset highlighting the chemical complementarity; the GTX molecule (S-Hexylglutathione, green sticks) is shown nestled within a solid potential surface, where pink and green regions represent donor and acceptor sites, respectively. (**c**) Lower inset detailing the interaction profile; the residual transparent surface reveals a complex stabilization network primarily through conventional hydrogen bonds with residues Tyr113, Ala10, and Arg06, complemented by a significant attractive charge interaction with Arg06. A dense network of van der Waals contacts (Ser65, Pro11, Ile52, Leu6, Gly8, Ser9, Met101, Leu33, Phe203, and Phe207) ensures steric complementarity, despite localized electrostatic repulsions with Glu64 and Tyr105. Dashed lines illustrate the binding interactions.

Regarding GST, the botanical compounds showed a marked capacity for hydrophobic occupancy within the catalytic domain (Figure 7). δ-Cadinene exhibited the highest binding stability (−6.6 kcal/mol, 14.50 Ki µM), followed by germacrene D (−6.4 kcal/mol, 20.5 Ki µM), both outperforming the reference inhibitor GTX (−6.0 kcal/mol, 40.0 Ki µM) and α-cypermethrin (−6.2 kcal/mol, 28.60 Ki µM).

The reference ligand GTX (Table 8) was anchored within the active site (G/H-sites) primarily through conventional hydrogen bonds with residues Tyr113, Ala10, and Arg06, alongside a network of van der Waals contacts. Visual analysis confirmed that δ-cadinene and germacrene D deeply embed into the xenobiotic binding site (H-site).

**Figure 8:**
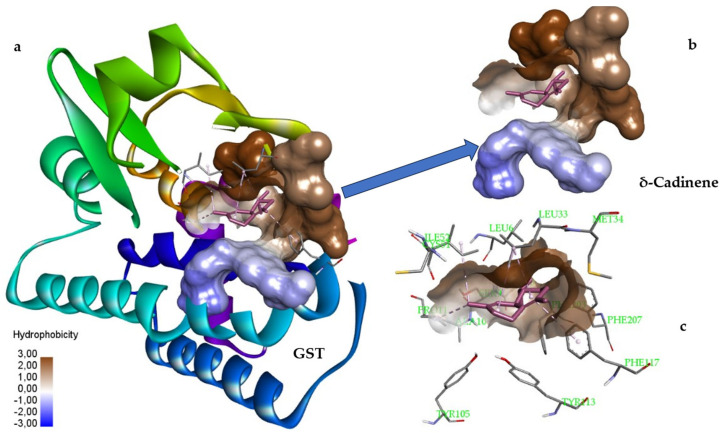
(**a**) Overview of the GST enzyme in ribbon representation (color gradient from N-terminus to C-terminus), highlighting 55 the hydrophobicity surface mapping at the xenobiotic binding site (H-site) (scale: brown = hydrophobic; blue = hydrophilic). The arrow indicates the transition to the magnified view of the binding pocket. (**b**) Upper inset showcasing the steric fit; the δ-cadinene molecule is shown anchored within the enzymatic cavity, presenting a favorable binding free energy and occupying the apolar domain required for catalytic activity. (**c**) Lower inset detailing the interaction profile; the residual transparent hydrophobicity surface reveals a stabilization network dominated by alkyl-type contacts with residues Leu33, Ile52, and Pro11, alongside a pi-alkyl interaction with Phe117. The stability of the complex is further supported by van der Waals interactions with Phe203, Phe207, Met34, Leu6, Gly8, and Ser9. Dashed lines illustrate the binding interactions.

**Figure 9:**
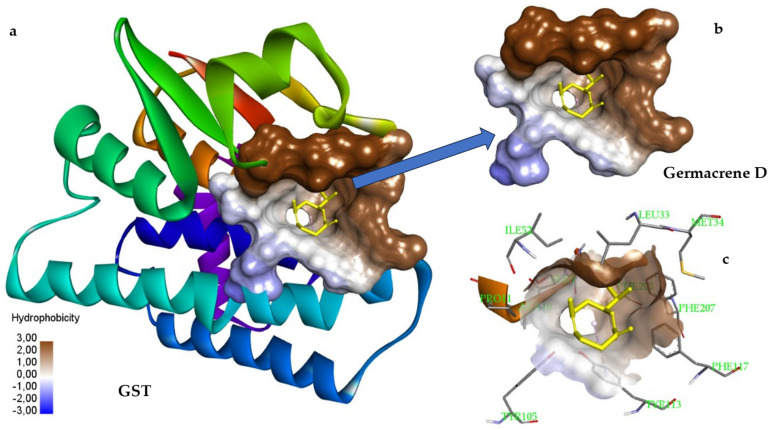
(**a**) Overview of the GST enzyme in ribbon representation (color gradient from N-terminus to C-terminus), highlighting the hydrophobicity surface mapping at the H-site (scale: brown = hydrophobic; blue = hydrophilic). The arrow indicates the transition to the magnified view of the binding pocket. (**b**) Upper inset showcasing the steric fit; the Germacrene D molecule (yellow sticks) is shown deeply embedded within the enzymatic cavity, presenting a favorable binding free energy and demonstrating significant complementarity with the apolar H-site domain. (**c**) Lower inset detailing the interaction profile; the residual transparent hydrophobicity surface reveals a binding mode primarily stabilized by a pi-alkyl interaction with Tyr113. The complex is further anchored by an extensive network of van der Waals interactions involving Phe117, Tyr105, Phe203, Phe207, Leu33, Ile52, Pro11, and Met34. Dashed lines illustrate the specific binding contacts.

The δ-cadinene complex was stabilized by alkyl-type contacts with Leu33, Ile52, and Pro11, alongside a pi-alkyl interaction with Phe117 (Figure 8). Germacrene D was anchored primarily by a pi-alkyl interaction with Tyr113 and an extensive network of van der Waals interactions involving Phe203, Phe207, and Met34 (Figure 9).

**Figure 10:**
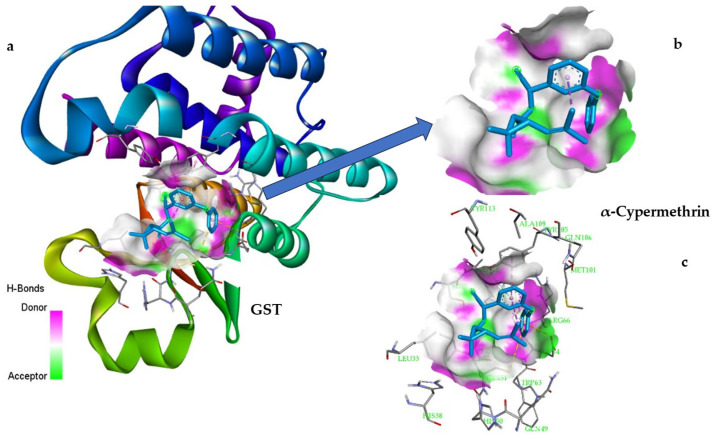
(**a**) Overview of the GST enzyme in ribbon representation (color gradient from N-terminus to C-terminus), highlighting the Hydrogen Bond (H-bond) surface mapping at the active site (scale: magenta = hydrogen bond donor; green = hydrogen bond acceptor). The arrow indicates the transition to the magnified views of the binding region. (**b**) Steric fit focus demonstrating the deep embedding of α-cypermethrin (cyan sticks) into the enzymatic cavity, which exhibits highly specific electrostatic complementarity with the localized H-bond donor (magenta) and acceptor (green) regions. (**c**) Comprehensive interaction profile detailing a complex stabilization network primarily anchored by specific H-bond interactions. The visualization highlights the conventional hydrogen bond with Ile52 (2.04 Å). The binding is further fortified by a diverse array of non-covalent contacts: a pi-donor hydrogen bond and pi-pi T-shaped interactions with Tyr105; pi-sulfur interaction with Cys51; and a pi-anion contact with Glu64. An extensive network of van der Waals interactions involving Met101, Arg66, Gln49, Gln106, His50, Leu33, Pro53, Trp63, and Tyr113 provides additional stabilization. Dashed lines and overlapping surfaces illustrate the specific binding contacts.

In contrast, α-cypermethrin (Figure 10) stabilization was anchored by a conventional hydrogen bond with Ile52 (2.04 Å), pi-sulfur interaction with Cys51, and pi-anion contact with Glu64. The high affinity of the sesquiterpenes for the apolar H-site reinforces their role as potent competitive inhibitors.

## 4. Discussion

The essential oil yield of *P. humillimum* (3.5 ± 0.4%) represents a significant production of secondary metabolites, consistent with the high metabolic plasticity reported for the Piperaceae family [54]. Chemical characterization revealed a profile overwhelmingly dominated by sesquiterpene hydrocarbons (91.47%), a characteristic that aligns with several Amazonian *Piper* species, such as *P. purusanum* C.DC. and *P. alatipetiolatum* Yuncker, which have been reported to yield 4.2% and 7.6% of essential oils containing 97.77% and 100% sesquiterpenes, respectively [32,36].

This yield and chemical composition are influenced by a complex interplay of biotic and abiotic factors, including seasonality, soil composition, and the specific phenological stage of the plant, all of which modulate biosynthetic pathways within the secretory structures, as previously detailed for other aromatic species such as *Tetradenia riparia* (Hochst.) Codd (Lamiaceae) [55].

From a structural perspective, the biological efficacy of the essential oil is likely dictated by the diverse configurations of its main sesquiterpenes [56]. Germacrene D and germacrene B are ten-membered carbocyclic sesquiterpenes characterized by flexible macrocyclic rings and systems of non-conjugated double bonds, which this structural flexibility allows for a high degree of conformational adaptability when interacting with biological targets [57].

In contrast, the essential oil contains a group of more rigid bicyclic and tricyclic structures, such as δ-cadinene and γ-muurolene, which possess decalin-type skeletons, featuring specific spatial arrangements of their isopropyl groups and double bonds that offer less conformational freedom [58]. Regarding the individual substances, the superior larvicidal potency of δ-cadinene observed in the bioassays, particularly against *An. darlingi*, can be attributed to its rigid bicyclic framework [59].

Other significant substances that further contribute to this structural complexity are β-cubebene that presents a rigid tricyclic framework containing a cyclopropane ring, while β-caryophyllene is distinguished by a rare fused cyclobutane ring, which imposes significant steric constraints [60]. Additionally, the presence of γ-elemene, a monocyclic sesquiterpene with a vinyl cyclohexane scaffold, and isobutyl acetate, an oxygenated aliphatic ester, expands the chemical diversity beyond the hydrocarbon dominance [61].

These structural characteristics, ranging from the high conformational adaptability of flexible macrocycles to the steric constraints of rigid bicyclic and tricyclic skeletons, combined with the high lipophilicity and unsaturated nature of the constituents identified in the essential oil of *P. humillimum*, are critical factors underpinning the larvicidal activity observed against *Ae. aegypti* and *An. darlingi* [62]. This molecular diversity is instrumental in the essential oil mode of action, as it facilitates efficient partitioning into the lipid-rich membranes of the larvae and ensures a broad range of hydrophobic interactions within the catalytic pockets of target enzymes [63].

It is worth noting that the natural volatility of essential oils and sesquiterpenes typically results in a shorter residual effect in the environment. This characteristic leads to reduced environmental persistence and a lower risk of long-term bioaccumulation, which are highly desirable traits for the development of sustainable botanical bioinsecticides, as widely reported in the literature, in contrast, conventional synthetic neurotoxicants, such as pyrethroids, are documented to maintain prolonged residual (up to 120 days) activity in aquatic bodies [28,64].

According to the literature, this extended persistence can cause severe non-target mortality in various aquatic vertebrates and invertebrates regardless of their ecological roles, affecting organisms such as *Chironomus riparius* Meigen, 1804 (Chironomidae), *Ceriodaphnia dubia* Richard, 1894 (Daphniidae), *Limnephilus lunatus* Curtis, 1834 (Limnephilidae), and *Astyanax bimaculatus* (Linnaeus, 1758) (Characidae) (Characidae) [11]. Therefore, the shorter residual effect observed for the *P. humillimum* essential oil indicates a lower potential for cumulative ecological impact compared to persistent synthetic alternatives [59].

Despite this transient environmental presence, the rapid cuticular penetration of these botanical compounds triggers an immediate biochemical collapse within the larva [12]. This is evidenced by a marked surge in H_2_O_2_ levels in both vectors following exposure to essential oil from *P. humillimum*, its major substances, and the synthetic insecticide a clear indication that the pro-oxidant effect of these derivatives overwhelmed the endogenous antioxidant capacity and induced a state of acute oxidative stress [65]. Corroborating this finding, a similar elevation in H_2_O_2_ levels (350 to 494 μmol H_2_O_2_ min^−1^ mg^−1^ protein) has been reported in *A. aegypti* larvae treated with the essential oils of *P. tuberculatum* Jacq., *P. brachypetiolatum* Yuncker, germacrene D, β-caryophyllene [54,66].

This redox imbalance subsequently led to extensive oxidative damage to vital macromolecules, specifically through lipid peroxidation and protein carbonylation. The induction of lipid peroxidation represents a critical event; the attack of reactive oxygen species on polyunsaturated fatty acids leads to the loss of membrane fluidity and integrity [67]. This disruption compromises the selective permeability of the cell membrane, resulting in the leakage of cytoplasmic contents and the collapse of electrochemical gradients essential for cellular homeostasis [68].

Simultaneously, the increase in protein carbonyl content serves as a hallmark of irreversible oxidative modification to amino acid side chains [69]. Such carbonylation, particularly at catalytic or allosteric sites, triggers a loss of biological function and renders proteins highly susceptible to premature proteolysis [70].

Collectively, these oxidative events drive the larval tissues towards both necrosis and apoptosis, leading to irreversible cellular death and, consequently, the high mortality rates recorded for *Ae. aegypti* and *An. darlingi* larvae [71]. This mechanism is supported by similar findings in the literature; for instance, high levels of lipid peroxidation (15.07 to 24.69 nmol MDA mg^−1^ protein) and protein carbonylation (4.2893 × 10^−3^ to 14.6278 × 10^−3^ nmol carbonyls mg^−1^ protein) were accompanied by mitochondrial dysfunction and severe damage to the midgut and Malpighian tubules of *A. aegypti* larvae after exposure to essential oil from *P. baccans* (Miq.) C.DC. [63], extract from *Xylaria* sp. (Xylariaceae) [10], and α-terthienyl [72].

To counteract this oxidative onslaught and mitigate the proliferation of reactive oxygen species, specifically H_2_O_2_, the larval antioxidant system is rapidly mobilized [10]. This defensive effort was evidenced in our study by the significantly elevated activities of SOD, which serves as the primary enzymatic line of defense, specifically neutralizing the highly reactive superoxide radical (O_2_^•−^) and converting it into H_2_O_2_ [73].

Although H_2_O_2_ is less reactive than the O_2_^•−^, it acts as a critical signaling molecule that triggers the subsequent activation of CAT, whose role is to detoxify H_2_O_2_ into water and molecular oxygen, thereby preventing its accumulation to toxic levels [73]. However, despite the marked induction of both enzymes in larvae exposed to essential oil from *P. humillimum*, germacrene D, *δ*-cadinene, and *α*-cypermethrin, the antioxidant machinery appeared to be overwhelmed [67].

The persistent and excessive production of H_2_O_2_ likely exceeds the catalytic capacity of the available CAT, leading to a systemic overload. This failure to maintain redox homeostasis suggests that the pro-oxidant pressure exerted by essential oils and substances surpassed the larvae’s compensatory mechanisms [65]. Consequently, the unchecked accumulation of H_2_O_2_ was allowed to proceed, fuelling the extensive lipid peroxidation and protein carbonylation previously described, and ultimately sealing the fate of the larvae [74].

Beyond essential oils, such as *P. cyrtopodon* (Miq.) C.DC. [75], increased activities of SOD (14.63 to 157.12 U mg^−1^ protein) and CAT (0.31 to 94.67 µmol H_2_O_2_ min^−1^ mg^−1^ protein) have also been identified in *Ae. aegypti*, *An. darlingi*, *Drosophila melanogaster* Meigen, 1830 (Drosophilidae), *Partamona helleri* Friese, 1900 (Apidae) following exposure to several substances including thiamethoxam [70], imidacloprid [44], 6-ishwarone [53].

In addition, the antioxidant imbalance previously discussed, the inhibition of GST represents a catastrophic failure in the larval defensive strategy, as this enzyme is essential not only for Phase II detoxification via glutathione (GSH) conjugation but also as a critical secondary antioxidant [8]. By functioning as a non-selenium glutathione peroxidase, GST neutralizes toxic lipid hydroperoxides and reactive aldehydes generated during membrane degradation, meaning its suppression by essential oil from *P. humillimum* and its substances effectively dismantled the larvae’s ability to terminate the autocatalytic chain of lipid peroxidation [10].

This enzymatic impairment created a lethal synergy with the observed oxidative stress because while H_2_O_2_ accumulation initiated cellular damage, the absence of functional GST allowed lipid-derived radicals to proliferate unchecked [67]. This lack of protective intervention prevented cellular stabilization, leading to an irreversible feedback loop of macromolecular degradation, and consequently, the inhibition of GST acted as a primary metabolic failure, leaving *Ae. aegypti* and *An. darlingi* larvae are biochemically defenseless and driving tissues towards systemic necrosis and death [44].

The susceptibility of GST to natural products is well-documented, with significant inhibition (0.2 to 1.2 µmol min^−1^ mg^−1^ protein) previously identified in *A. stephensi* Liston, 1901 (Culicidae) and *Rhipicephalus microplus* (Canestrini, 1888) (Ixodidae) following treatment with extracts from *Annona muricata* L. (Annonaceae), *Carica papaya* L. (Caricaceae), *Cinnamomum zeylanicum* J. Presl (Lauraceae), *Phyllanthus emblica* L. (Phyllanthaceae), *Punica granatum* L. (Lythraceae), *Syzygium cumini* (L.) Skeels (Myrtaceae) among others [16]. These findings corroborate our results, that botanical secondary metabolites frequently target the GST catalytic site, thereby neutralizing a vital line of defense against both xenobiotics and oxidative damage [75].

In contrast to the inhibitory effect observed for GST, the increased activities of MFO and both α- and β-esterases indicate that these enzyme families treated the essential oil from *P. humillimum* and its major substances as substrates rather than inhibitors [15]. MFOs initiate Phase I detoxification by introducing polar groups into lipophilic xenobiotics, while esterases hydrolyse ester bonds to facilitate their elimination [16].

This upregulation represents an active, yet insufficient, metabolic attempt by *Ae. aegypti* and *An. darlingi* larvae to neutralize the encroaching botanical toxins [76]. Our findings align with the increased activities of MFO (26.67 to 55.00 nmol cyt c min^−1^ mg^−1^ protein) and α- and β-esterases (29.33 to 81.67 µmol min^−1^ mg^−1^ protein) were also identified in these vector larvae after exposure to extrato metanólico de *P. purusanum*, piplartina [38].

However, this asymmetrical response created a lethal imbalance because the simultaneous impairment of the GST pathway prevented the completion of the detoxification cycle despite the mobilization of MFO and esterase systems [77]. This functional mismatch ensured that the larvae could neither fully biotransform the primary substances nor mitigate the resulting oxidative by-products, ultimately driving the tissues towards systemic necrosis and death [78].

Complementing the metabolic disruption, the significant inhibition of AChE acted as the primary neurotoxic trigger in the lethal cascade. AChE is responsible for the rapid hydrolysis of the neurotransmitter acetylcholine at synaptic junctions, and its suppression by the essential oil from *P. humillimum* and its substances leads to the overstimulation of cholinergic receptors [16]. This enzymatic failure results in continuous nerve impulse transmission, manifesting as the tremors, loss of coordination, and eventual paralysis observed in *Ae. aegypti* and *An. darlingi* larvae during the bioassays [65].

Our findings align with several studies reporting AChE inhibition (3.11 to 15 μmol min^−1^ mg^−1^ protein; IC_50_ from 4.11 to 37.08 µg/mL) as a key factor in the larvicidal activity of essential oils from *Rosmarinus officinalis* (L.) Schleid. (Lamiaceae) [65], *Piper divaricatum* G. Mey. (Piperaceae) [79], *Cymbopogon citratus* (DC) Stapf. (Poaceae) along with substances such as embelin [80], ishwarol B [37].

The simultaneous inhibition of both GST and AChE creates a synergistic lethal effect, while the larvae were biochemically disarmed against oxidative and xenobiotic stress, their neuromuscular control was also compromised [81]. Such dual-target action is a hallmark of potent botanical insecticides, where the essential oil components interfere with vital regulatory enzymes across different systems [82]. Consequently, the convergence of neurotoxicity and metabolic failure prevented any possibility of recovery, driving the larvae towards rapid systemic collapse and confirming the essential oil from *P. humillimum* as a multi-target larvicidal agent [15].

The molecular docking simulations provide a robust structural explanation for the potent enzymatic inhibition observed in our biochemical assays. The high binding affinities of the major substances for both GST and AChE binding sites suggest that these sesquiterpenes may act as high-affinity ligands capable of outperforming both natural substrates and commercial inhibitors [15].

However, it is important to note that these *in silico* results serve as a supportive structural rationale and hypothesis-generating framework rather than a definitive physical confirmation of the mechanism of action. An inherent limitation of this computational approach is the reliance on enzyme structures from related model mosquito species such as *An. gambiae* for the Culicidae models due to the current absence of high-resolution crystallographic targets specifically for *Ae. aegytpi* or *An. darlingi* in public databases [51]. Nevertheless, because these catalytic domains and target residues are highly conserved among culicids, and because the computational binding energies closely mirror the severe enzymatic inhibition observed in our experimental assays, these models represent a reliable framework for understanding the interaction dynamics at the active sites [52].

Regarding GST, the deep embedding of these common sesquiterpenes into the Hydrophobic Pocket (H-site) clarifies the metabolic collapse observed. Both ligands demonstrated a shared binding pattern, anchoring to key residues such as Tyr113, Phe117, Leu33, and Ile52 [23]. Since the H-site is specifically designed to bind lipophilic xenobiotics for conjugation with glutathione, the high occupancy of these specific residues by δ-cadinene and germacrene D directly prevents the enzyme from neutralizing oxidative by-products [83].

The prevalence of hydrophobic interactions, such as alkyl and pi-alkyl contacts with these common residues, ensures the formation of a stable and persistent complex that effectively disarms the primary chemical defense of the larvae [84].

In the case of AChE, the spatial orientation of these substances within the peripheral anionic site is equally consistent. Both common ligands targeted the same strategic residues at the entrance of the catalytic gorge, including Tyr282, Phe490, and Tyr493, acting as molecular plugs that obstruct the passage of acetylcholine [85].

The AChE model used in this study (PDB ID: 6ARY) corresponds to the G119S mutant, a variant associated with high levels of insecticide resistance, including pyrethroids [17]. The fact that δ-cadinene and germacrene D exhibited superior binding potential compared to α-cypermethrin while interacting with these conserved residues suggests that these substances may overcome established resistance mechanisms [18]. This mechanism of competitive inhibition by obstruction ensures a continuous nerve impulse transmission that leads to the rapid onset of neurotoxic symptoms [50].

The convergence of these structural findings with *in vivo* and *in vitro* data reinforces the status of the essential oil from *P. humillimum* as a multi-target larvicidal agent [84]. The presence of these high-affinity ligands as common major substances, sharing identical molecular targets within the enzymes, ensures that the essential oil can simultaneously compromise antioxidant protection and neuromuscular signaling, driving the larvae towards an irreversible systemic collapse [84].

## 5. Conclusions

The essential oil from *P. humillimum*, δ-cadinene and germacrene D, have larvicidal action caused by systemic failure triggered by acute oxidative stress and irreversible cellular damage. While certain metabolic pathways are recruited in response to treatment, the direct inhibition of key enzymes in neurotransmission and detoxification, supported by high-affinity binding at catalytic sites, is decisive for larval mortality.

Molecular docking confirms that the binding stability of these sesquiterpenes surpasses that of commercial standards, even against resistant mutants. Despite their natural volatility reducing residual persistence compared to synthetic pyrethroids, their high initial efficacy and multi-target action highlight their potential as sustainable alternatives for vector management. These findings provide robust evidence for using the essential oil form *P. humillimum* to control *Ae. aegypti* and *An. darlingi* larvae populations.

## Figures and Tables

**Table 1:** Chemical composition of essential oil from *P. humillimum* leaves.

Compound	Area (%) ^a^	RI ^b^	RI ^c^	Identification	Chemical Class
Isobutyl acetate	6.71	752	757	RI, MS	Aliphatic ester
β-Cubebene	8.36	1379	1388	RI, MS	Sesquiterpene
γ-Elemene	1.81	1391	1402	RI, MS	Sesquiterpene
β-Caryophyllene	1.07	1419	1419	RI, MS	Sesquiterpene
γ-Muurolene	1.04	1471	1479	RI, MS	Sesquiterpene
Germacrene D	61.51	1483	1486	RI, MS	Sesquiterpene
δ-Cadinene	17.46	1520	1523	RI, MS	Sesquiterpene
Germacrene B	2.04	1554	1561	RI, MS	Sesquiterpene

^a^ Relative area calculated from the peak area relative to the total peak area in the GC-FID chromatogram. ^b^ RI retention index calculated using *n*-alkanes (C_8_–C_30_) on the TR5 column. ^c^ Retention index literature. MS mass spectra compared with the literature.

**Table 2:** LC_50_ and CL_90_ of the essential oil, germacrene D, and δ-cadinene from *P. humillimum* against *Ae. aegypti* and *An. darlingi* larva.

Sample	Larvae	LC_50_ (µg/mL) (LCL-UCL)	LC_90_ (µg/mL) (LCL-UCL)	χ^2^ (Df)	*p*-Value	Linear Regression
Essential oil	*Ae. aegypti*	37.42 (34.489–40.280) ^a^	70.41 (64.475–78.400) ^a^	3.984 (3)	0.263	Y = −7.345 + 4.669
Germacrene D		46.04 (42.9020–49.134) ^b^	82.18 (75.618–91.094) ^a^	6.947 (3)	0.074	Y = −8.473 + 5.094
δ-Cadinene		39.88 (36.671–43.004) ^ab^	79.15 (71.998–88.999) ^a^	6.742 (3)	0.081	Y = −6.891 + 4.305
Essential oil	*An. darlingi*	42.50 (39.101–45.845) ^a^	86.97 (78.648–98.674) ^a^	7.245 (3)	0.064	Y = −6.711 + 4.121
Germacrene D		42.25 (39.117–45.323) ^a^	79.03 (72.403–88.025) ^a^	3.321 (3)	0.345	Y = −7664 + 4.713
δ-Cadinene		34.75 (31.621–37.761) ^a^	71.93 (65.173–81.243) ^a^	4.499 (3)	0.212	Y = −6.252 + 4.057

LC_50_ and _90_—Concentrations needed to kill 50 and 90% of larvae. LCL—Lower confidence limit of 95%. UCL—Upper confidence limit of 95%. χ^2^—Non significant Chi-square (*p* > 0.0001). Df—Degrees of freedom. Different letters (a to b) within the same column indicate statistically significant differences, as determined by one-way ANOVA and Tukey’s test (*F* (2, 6) = 6.326, *p* < 0.0333 for LC_50_ and *F* (2, 6) = 1892, *p* = 0.2307 for LC_90_) for *Ae. aegypti*. While *F* (2, 6) = 5.768, *p* < 0.0401 for LC_50_ and *F* (2, 6) = 5.768, *p* = 0.1791 for LC_90_ for *An. darlingi.* The positive control α-cypermethrin evaluated at 0.21 μg/mL resulted in 100% larvae mortality. No larvae died in the negative control (DMSO) (20 to 100 µg/mL).

**Table 3:** *In vivo* Hydrogen peroxide (H_2_O_2_) production, Lipid oxidative damage, and Protein oxidative damage *Ae. aegypti* and *An. darlingi* larvae induced by the essential oil from *P. humillimum* and its major substances.

	Sample	*Ae. aegypti* (Mean ± SD)	*F* (DFn, DFd)	*p*-Value	*An. darlingi* (Mean ± SD)	*F* (DFn, DFd)	*p*-Value
H_2_O_2_ production (µmol H_2_O_2_ min^−1^ mg^−1^ protein)	Essential oil	61.00 ± 5.03 ^a^	4, 10 = 92.75	0.0001	74.00 ± 7.01 ^a^	4, 10 = 208.8	0.0001
	Germacrene D	36.67 ± 1.52 ^b^			49.33 ± 1.52 ^b^		
	δ-Cadinene	55.67 ± 6.11 ^a^			64.00 ± 3.60 ^c^		
	*α*-Cypermethrin	82.00 ± 3.60 ^c^			81.33 ± 1.52 ^a^		
	DMSO	5.66 ± 2.51 ^d^			4.00 ± 1.05 ^d^		
Lipid oxidative damage (nmol MDA mg^−1^ protein)	Essential oil	54.00 ± 5.10 ^a^	4, 10 = 113.9	0.0001	42.33 ± 7.63 ^a^	4, 10 = 124.6	0.0001
	Germacrene D	43.3 ± 6.02 ^a^			44.67 ± 2.51 ^a^		
	δ-Cadinene	47.35 ± 4.04 ^a^			54.00 ± 4.35 ^a^		
	*α*-Cypermethrin	59.61 ± 2.01 ^b^			81.67 ± 3.05 ^b^		
	DMSO	1.33 ± 0.33 ^c^			3.33 ± 1.52 ^c^		
Protein oxidative damage (nmol carbonyls mg^−1^ protein)	Essential oil	74.67 ± 6.02 ^a^	4, 10 = 193.4	0.0001	60.67 ± 3.78 ^ab^	4, 10 = 293.9	0.0001
	Germacrene D	61.67 ± 6.80 ^b^			58.33 ± 2.30 ^a^		
	δ-Cadinene	83.00 ± 2.64 ^a^			68.00 ± 3.02 ^b^		
	*α*-Cypermethrin	77.33 ± 3.51 ^a^			86.61 ± 4.50 ^c^		
	DMSO	3.00 ± 1.01 ^d^			2.01 ± 0.1 ^d^		

Different letters (a–d) within the same column indicate statistically significant differences, as determined by one-way ANOVA followed by Tukey’s test (*p* < 0.05). *F* (DFn, DFd): Represents the *F*-statistic ratio, where DFn and DFd correspond to the degrees of freedom for the numerator (between-group variance) and the denominator (residual error variance), respectively.

**Table 4:** *In vivo* Superoxide dismutase and Catalase activities in *Ae. aegypti* and *An. darlingi* larvae induced by the essential oil from *P. humillimum* and its major substances.

	Sample	*Ae. aegypti* (Mean ± SD)	*F* (DFn, DFd)	*p*-Value	*An. darlingi* (Mean ± SD)	*F* (DFn, DFd)	*p*-Value
SOD activity (U mg^−1^ protein)	Essential oil	87.33 ± 11.59 ^a^	4, 10 = 102.9	0.0001	90.00 ± 2.64 ^ab^	4, 10 = 661.7	0.0001
	Germacrene D	83.31 ± 6.80 ^a^			85.31 ± 4.04 ^a^		
	δ-Cadinene	83.41 ± 2.01 ^a^			95.00 ± 3.60 ^b^		
	*α*-Cypermethrin	92.41 ± 3.21 ^a^			104.00 ± 1.06 ^c^		
	DMSO	4.69 ± 0.12 ^b^			1.33 ± 0.52 ^d^		
CAT activity (µmol H_2_O_2_ min^−1^ mg^−1^ protein)	Essential oil	74.31 ± 7.02 ^a^	4, 10 = 166.0	0.0001	82.09 ± 1.03 ^a^	4, 10 = 599.9	0.0001
	Germacrene D	79.00 ± 9.16 ^a^			80.41 ± 2.08 ^a^		
	δ-Cadinene	89.61 ± 2.01 ^a^			77.61 ± 5.29 ^a^		
	*α*-Cypermethrin	107.07 ± 3.05 ^b^			103.00 ± 2.02 ^b^		
	DMSO	1.33 ± 0.1 ^c^			0.66 ± 0.01 ^c^		

Different letters (a–d) within the same column indicate statistically significant differences, as determined by one-way ANOVA followed by Tukey’s test (*p* < 0.05). *F* (DFn, DFd): Represents the *F*-statistic ratio, where DFn and DFd correspond to the degrees of freedom for the numerator (between-group variance) and the denominator (residual error variance), respectively.

**Table 5:** *In vivo* Glutathione S-transferase, Mixed-Function Oxidase (MFO), and α-esterase activities in *Ae. aegypti* and *An. darlingi* larvae induced by the essential oil from *P. humillimum* and its major substances.

	Sample	*Ae. aegypti* (Mean ± SD)	*F* (DFn, DFd)	*p*-Value	*An. darlingi* (Mean ± SD)	*F* (DFn, DFd)	*p*-Value
GST activity (µmol min^−1^ mg^−1^ protein)	Essential oil	11.01 ± 2.01 ^a^	4, 10 = 167.0	0.0001	15.31 ± 3.19 ^a^	4, 10 = 316.8	0.0001
	Germacrene D	15.67 ± 1.52 ^a^			15.67 ± 1.52 ^ab^		
	δ-Cadinene	11.67 ± 1.52 ^a^			9.67 ± 3.05 ^ab^		
	*α*-Cypermethrin	7.89 ± 1.29 ^a^			8.14 ± 2.51 ^b^		
	DMSO	67.61 ± 5.89 ^b^			70.67 ± 3.21 ^c^		
MFO activity (nmol cyt c min^−1^ mg^−1^ protein)	Essential oil	63.67 ± 6.42 ^a^	4, 10 = 59.53	0.0001	59.33 ± 7.50 ^a^	4, 10 = 103.2	0.0001
	Germacrene D	59.61 ± 5.03 ^a^			70.21 ± 3.21 ^a^		
	δ-Cadinene	61.17 ± 11.37 ^a^			73.52 ± 6.42 ^a^		
	*α*-Cypermethrin	73.67 ± 6.42 ^a^			71.61 ± 3.51 ^a^		
	DMSO	3.33 ± 1.16 ^b^			5.33 ± 2.08 ^c^		
α-esterase activity (µmol min^−1^ mg^−1^ protein)	Essential oil	38.41 ± 4.04 ^a^	4 10 = 32.64	0.0001	42.67 ± 1.52 ^a^	4, 10 = 59.81	0.0001
	Germacrene D	56.61 ± 10.79 ^ab^			61.31 ± 9.29 ^a^		
	δ-Cadinene	43.67 ± 12.86 ^a^			59.67 ± 3.05 ^a^		
	*α*-Cypermethrin	74.64 ± 4.04 ^b^			81.01 ± 4.35 ^b^		
	DMSO	8.01 ± 1.12 ^c^			2.31 ± 0.04 ^c^		

Different letters (a–c) within the same column indicate statistically significant differences, as determined by one-way ANOVA followed by Tukey’s test (*p* < 0.05). *F* (DFn, DFd): Represents the *F*-statistic ratio, where DFn and DFd correspond to the degrees of freedom for the numerator (between-group variance) and the denominator (residual error variance), respectively.

**Table 6:** *In vivo* α-esterase and acetylcholinesterase (AChE) activities in *Ae. aegypti* and *An. darlingi* larvae induced by the essential oil from *P. humillimum* and its major substances.

	Sample	*Ae. aegypti* (Mean ± SD)	*F* (DFn, DFd)	*p*-Value	*An. darlingi* (Mean ± SD)	*F* (DFn, DFd)	*p*-Value
β-esterase (µmol min^−1^ mg^−1^ protein)	Essential oil	42.67 ± 3.05 ^a^	4, 10 = 412.4	0.0001	30.33 ± 2.08 ^a^	4, 10 = 295.0	0.0001
	Germacrene D	51.09 ± 1.52 ^b^			31.30 ± 1.15 ^a^		
	δ-Cadinene	61.47 ± 3.05 ^c^			44.00 ± 2.64 ^b^		
	*α*-Cypermethrin	76.61 ± 2.08 ^d^			65.29 ± 3.78 ^c^		
	DMSO	3.31 ± 1.52 ^e^			1.03 ± 0.95 ^d^		
AChE activity (μmol min^−1^ mg^−1^ protein)	Essential oil	14.67 ± 1.52 ^a^	4, 10 = 252.4	0.0001	17.00 ± 1.00 ^a^	4, 10 = 576.4	0.0001
	Germacrene D	14.33 ± 3.78 ^a^			18.31 ± 2.51 ^a^		
	δ-Cadinene	15.00 ± 1.00 ^a^			18.00 ± 1.00 ^a^		
	*α*-Cypermethrin	1.67 ± 0.01 ^b^			3.00 ± 1.00 ^b^		
	DMSO	86.00 ± 7.00 ^c^			78.01 ± 3.60 ^c^		

Different letters (a–e) within the same column indicate statistically significant differences, as determined by one-way ANOVA followed by Tukey’s test (*p* < 0.05). *F* (DFn, DFd): Represents the *F*-statistic ratio, where DFn and DFd correspond to the degrees of freedom for the numerator (between-group variance) and the denominator (residual error variance), respectively.

**Table 7:** *In silico* comparative analysis of the inhibitory potential of δ-cadinene and germacrene D against AChE (PDB ID: 6ARY), compared to NAG and α-cypermethrin.

Compound	Interacting Residues	Chain	Bond Length (Å)	Type of Interaction	Binding Energy (kcal/mol)	Inhibition Constant (Ki µM)	RMSD	Target Domain	Suggested Mechanism
NAG	Asn259	A	2.49	Conventional Hydrogen Bond	−5.6	78.0	0.6454	Distal/Allosteric Site	Indirect modulation via non-catalytic site binding
	Ser306	A	2.78	Conventional Hydrogen Bond					
	Leu288	A	3.26	Conventional Hydrogen Bond					
	Asn248	A	2.87	Conventional Hydrogen Bond					
	His293	A	2.38, 3.63, 3.80	Conventional Hydrogen Bond, Pi-Donor Hydrogen Bond, Pi-Donor Hydrogen Bond					
	Thr287	A	3.35, 3.66	Conventional Hydrogen Bond, Carbon Hydrogen Bond					
	Asp292	A	2.83	Carbon Hydrogen Bond					
	Asp289	A	3.76	Carbon Hydrogen Bond					
	Ala286	A	-	van der Waals					
	Tyr291	A	-	van der Waals					
	Leu192	A	-	van der Waals					
	Pro224	A	-	van der Waals					
	Thr249	A	-	van der Waals					
*δ*-Cadinene	Val235	A	5.34	Alkyl	−7.8	1.94		Peripheral Anionic Site	Competitive inhibition by obstructing the gorge entrance
	Tyr493	A	3.80, 4.86	Pi-Alkyl, Pi-Alkyl					
	Phe490	A	5.12	Pi-Alkyl					
	Tyr489	A	4.80	Pi-Alkyl					
	Asp233	A	-	van der Waals					
	Trp441	A	-	van der Waals					
	Tyr494	A	-	van der Waals					
	Tyr282	A	-	van der Waals					
	Ser280	A	-	van der Waals					
	Phe449	A	-	van der Waals					
Germacrene D	Tyr282	A	5.08	Pi-Alkyl	−7.9	1.63		Peripheral Anionic Site	Competitive inhibition by obstructing the gorge entrance
	Trp441	A	4.67	Pi-Alkyl					
	Tyr493	A	3.91	Pi-Sigma					
	Tyr494	A	-	van der Waals					
	Glu448	A	-	van der Waals					
	Cys447	A	-	van der Waals					
	Gly445	A	-	van der Waals					
	Ile446	A	-	van der Waals					
	Phe449	A	-	van der Waals					
	Phe490	A	-	van der Waals					
	Tyr489	A	-	van der Waals					
	Ser280	A	-	van der Waals					
*α*-Cypermethrin	Val235	A	5.0	Alkyl	−6.8	10.40		Peripheral Anionic Site/Catalytic Gorge	Competitive inhibition by blocking access to the active site
	Phe490	A	-	van der Waals					
	Phe449	A	-	van der Waals					
	Cys447	A	-	van der Waals					
	Ile446	A	-	van der Waals					
	Tyr493	A	-	van der Waals					
	Tyr282	A	-	van der Waals					
	Tyr494	A	-	van der Waals					
	Trp441	A	-	van der Waals					
	Ile231	A	-	van der Waals					

NAG: 2-Acetamido-2-deoxy-β-D-glucopyranose (reference ligand/inhibitor). RMSD = Root Mean Square Deviation. Binding mechanisms were classified based on the spatial orientation relative to the catalytic gorge.

**Table 8:** *In silico* comparative analysis of the inhibitory potential of δ-cadinene and germacrene D against GST (PDB ID: 1PN9), compared to GTX and α-cypermethrin.

Compound	Interacting Residues	Chain	Bond Length (Å)	Type of Interaction	Binding Energy (kcal/mol)	Inhibition Constant (Ki µM)	RMSD	Target Domain	Suggested Mechanism
GTX	Phe117	A	4.22, 5.08	Pi-Alkyl, Pi-Alkyl	−6.0	40	1.7153	Active Site (G/H-sites)	Substrate analog; direct catalytic binding
	Tyr113	A	4.66, 2.89	Pi-Alkyl, Conventional Hydrogen Bond					
	Ala10	A	2.97	Conventional Hydrogen Bond					
	Arg06	A	5.41, 3.18	Conventional Hydrogen Bond, Attractive Charge					
	Glu64	A	5.24	Unfavorable Negative–Negative					
	Tyr105	A	2.19	Unfavorable Donor–Donor					
	Ser65	A	-	van der Waals					
	Pro11	A	-	van der Waals					
	Ile52	A	-	van der Waals					
	Leu6	A	-	van der Waals					
	Gly8	A	-	van der Waals					
	Ser9	A	-	van der Waals					
	Met101	A	-	van der Waals					
	Leu33	A	-	van der Waals					
	Phe203	A	-	van der Waals					
	Phe207	A	-	van der Waals					
*δ*-Cadinene	Leu33	A	4.49, 5.07	Alkyl, Alkyl	−6.6	14.50		Hydrophobic Pocket (H-site)	Competitive inhibition via hydrophobic occupancy
	Ile52	A	4.97	Alkyl					
	Pro11	A	4.93	Alkyl					
	Phe117	A	5.24	Pi-Alkyl					
	Phe203	A	-	van der Waals					
	Phe207	A	-	van der Waals					
	Met34	A	-	van der Waals					
	Leu6	A	-	van der Waals					
	Gly8	A	-	van der Waals					
	Ser9	A	-	van der Waals					
Germacrene D	Tyr113	A	4.67	Pi-Alkyl	−6.4	20.5		Hydrophobic Pocket (H-site)	Competitive inhibition via hydrophobic occupancy
	Tyr105	A	-	van der Waals					
	Ile52	A	-	van der Waals					
	Pro11	A	-	van der Waals					
	Ser9	A	-	van der Waals					
	Ala10	A	-	van der Waals					
	Leu33	A	-	van der Waals					
	Gly8	A	-	van der Waals					
	Phe203	A	-	van der Waals					
	Phe207	A	-	van der Waals					
	Phe117	A	-	van der Waals					
*α*-Cypermethrin	Ile52	A	2.04	Conventional Hydrogen Bond	−6.2	28.60		H-site/Interface	Competitive inhibition anchored by a specific H-bond
	Tyr105	A	3.28. 5.19	Pi-Donor Hydrogen Bond, Pi-Pi T-shaped					
	Pro11	A	5.07	Pi-Alkyl					
	Cys51	A	5.56	Pi-Sulfur					
	Glu64	A	3.82	Pi-Anion					
	Ser65	A	-	van der Waals					
	Arg66	A	-	van der Waals					
	Pro53	A	-	van der Waals					
	Tyr113	A	-	van der Waals					
	Ser9	A	-	van der Waals					
	Ala10	A	-	van der Waals					
	Leu33	A	-	van der Waals					
	His38	A	-	van der Waals					
	His50	A	-	van der Waals					
	Gln49	A	-	van der Waals					

GTX: S-Hexylglutathione (reference ligand/inhibitor). RMSD = Root Mean Square Deviation. H-site: Hydrophobic pocket; G-site: Glutathione binding site.

## Data Availability

Data will be made available on request.

## Supplementary Materials

The following supporting information can be downloaded at: https://www.mdpi.com/article/10.3390/molecules31111960/s1, Table S1: Configuration parameters for the molecular docking of *P. humillimum* substances and α-cypermethrin against AChE and GST targets with details the grid box coordinates (center and dimensions), energy range, and exhaustiveness settings employed for each ligand-protein interaction; Figure S1: Confocal laser scanning microscopy (CLSM) of *Ae. aegypti* larvae treated with essential oil from *P. humillimum*. (A) Cephalic region showing acute accumulation of the essential oil (high fluorescence intensity) in the oral cavity and feeding apparatus. (B) Longitudinal section of the midgut exhibiting severe disruption of the brush border integrity, accompanied by extensive cytoplasmic vacuolization (arrowheads), indicating cellular stress. (C) Thoracic region highlighting congestion and structural damage to the tubular excretory system. (D) and (F) Posterior segments showing fragmented fluorescence patterns, potentially representing apoptotic nuclei or chromatin condensation. (E) Lateral view of the abdomen demonstrating significant exo-cuticular damage and loss of chitinous layer uniformity. (G) Detail of the abdominal midgut showing advanced disintegration of the intestinal epithelium and the presence of localized fluorescent clusters. (H) Anal papillae and terminal segment displaying altered morphology and irregular distribution of the essential oil within the anal saddle; Figure S2: Morphological and histopathological alterations in *Ae. aegypti* larvae observed by light microscopy after exposure to essential oil from *P. humillimum*. (A) Head capsule displaying pronounced internal tissue darkening and loss of structural definition in the cephalic region. (B) Thoracic segment showing severe narrowing of the anterior midgut and initial signs of peritrophic matrix degradation. (C) Detail of the head highlighting localized necrotic foci (dark pigmented spots) near the ocular and feeding apparatus. (D) Prothoracic region exhibiting marked tissue contraction and significant disorganization of the internal architecture. (E) Anterior midgut demonstrating the hallmark of cytotoxicity: extensive detachment of the intestinal epithelium from the basement membrane, resulting in a constricted central canal. (F–G) Posterior abdominal segments displaying massive tissue melanization and darkening along the alimentary canal, indicative of advanced systemic necrosis. (H) Terminal segment and anal papillae showing altered morphology, including cellular swelling and loss of transparency, suggesting a complete collapse of osmoregulatory capacity. Scale bars: 100 µm. The observed darkening, epithelial shedding, and organ deformation collectively indicate severe cytotoxic and neurotoxic effects leading to rapid larval mortality; Figure S3: Effects of the essential oil from *P. humillimum* along with germacrene D, δ-cadinene, and α-cypermethrin on GST activity in (a) *Ae. aegypti* and (b) *An. darlingi*. DMSO was employed as the control group are expressed as mean ± standard deviation. Statistical significance is indicated as follows: ** (*p* < 0.01), *** (*p* < 0.001), **** (*p* < 0.0001), and ns (not significant) according to one-way ANOVA followed by Tukey’s post-hoc test; Figure S4: Effects of the essential oil from *P. humillimum* along with germacrene D, δ-cadinene, and α-cypermethrin on MFO activity in (a) *Ae. aegypti* and (b) *An. darlingi*. DMSO was employed as the control group. Data are expressed as mean ± standard deviation. Statistical significance is indicated as follows: ** (*p* < 0.01), *** (*p* < 0.001), **** (*p* < 0.0001), and ns (not significant) according to one-way ANOVA followed by Tukey’s post-hoc test; Figure S5: Effects of the essential oil from *P. humillimum* along with germacrene D, δ-cadinene, and α-cypermethrin on α-esterase activity in (a) *Ae. aegypti* and (b) *An. darlingi*. DMSO was employed as the control group. Data are expressed as mean ± standard deviation. Statistical significance is indicated as follows: * (*p* < 0.05), ** (*p* < 0.01), **** (*p* < 0.0001), and ns (not significant) according to one-way ANOVA followed by Tukey’s post-hoc test; Figure S6: Effects of the essential oil from *P. humillimum* along with germacrene D, δ-cadinene, and α-cypermethrin on β-esterase activity in (a) *Ae. aegypti* and (b) *An. darlingi.* DMSO was employed as the control group. Data are expressed as mean ± standard deviation. Statistical significance is indicated as follows: * (*p* < 0.05), ** (*p* < 0.01), **** (*p* < 0.0001), and ns (not significant) according to one-way ANOVA followed by Tukey’s post-hoc test; Figure S7: Effects of the essential oil from *P. humillimum*, germacrene D, δ-cadinene, and α-cypermethrin on AChE activity in (a) *Ae. aegypti* and (b) *An. darlingi*. DMSO was employed as the control group. Data are expressed as mean ± standard deviation. Statistical significance is indicated as follows: * (*p* < 0.05), *** (*p* < 0.001), **** (*p* < 0.0001), and ns (not significant) according to one-way ANOVA followed by Tukey’s post-hoc test [86].
