# Supplementary material for: Pioneering Investigation on the Larvicidal Mechanism and Chemical Profile of Piper humillimum C.DC. (Piperaceae) Essential Oil: Integrating In Vivo, In Vitro, and In Silico Models Against Aedes aegypti (Linnaeus, 1762) and Anopheles darlingi Root, 1926 (Culicidae)
Source: Molecules. 2026 Jun 4;31(11):1960. doi: 10.3390/molecules31111960 (PMC13258296; doi:10.3390/molecules31111960)
Supplement: Supplementary file 1 [file molecules-31-01960-s001.zip › molecules-4328270-supplementary.pdf]

Supplementary Material

Table S1. Configuration parameters for the molecular docking of *P. humillimum* substances and α-cypermethrin against AChE and GST targets with details the grid box coordinates (center and dimensions), energy range, and exhaustiveness settings employed for each ligand-protein interaction.

Enzyme	Substance	Center			Dimension			Number poses	Energy range	Exhaustiveness	Spacing (Å)
		x	y	z	x	y	z				
AChE	NAG*	-54.821	87,470	-6.033	126	126	126	50	50	50	1.000
	Germacrene D	- 39.692	57.884	-4.387							
	δ-Cadinene	-63.738	7.394	5.265							
	α-Cypermethrin	−54.065	56.185	56.000							
GST	GTX**	47.850	42.171	15.063							
	Germacrene D	41.044	35.422	15.063							
	δ-Cadinene	51.014	42.172	35.091							
	α-Cypermethrin	61.044	45.422	25.091							

NAG* 2-Acetamido-2-deoxy-beta-D-glucopyranose (Redocking ligand/Co-crystallised ligand). GTX** S-Hexylglutathione (Redocking ligand/Co-crystallised ligand. Spacing are expressed in Ångströms (Å).

Methodology

Optical and confocal microscopy

Larvae of *Ae. aegypti* were processed following a phalloidin staining protocol adapted from Neto et al. (2024), with modifications. Briefly, the samples were chemically fixed with 4% paraformaldehyde in PBS for 12 h. Subsequently, the samples were washed three times with PBS and permeabilized with 1% Triton X-100 at room temperature for 10 min, followed by two additional washes with PBS.

The larvae were then incubated with Alexa Fluor 488 Phalloidin in a dark chamber for 60 min. After incubation, 10 μL of ProLong Diamond Antifade Mountant with DAPI was added, and the samples were mounted between a glass slide and coverslip. The slides were allowed to cure for 12 h prior to imaging. Confocal images were acquired using a Leica TCS SP8 confocal microscope with LAS X software, using excitation wavelengths (λ) of 488 and 538 nm.

To complement the fluorescence data, the same slides were additionally examined under bright-field microscopy using a Leica DM4 B microscope to evaluate general larval morphology.

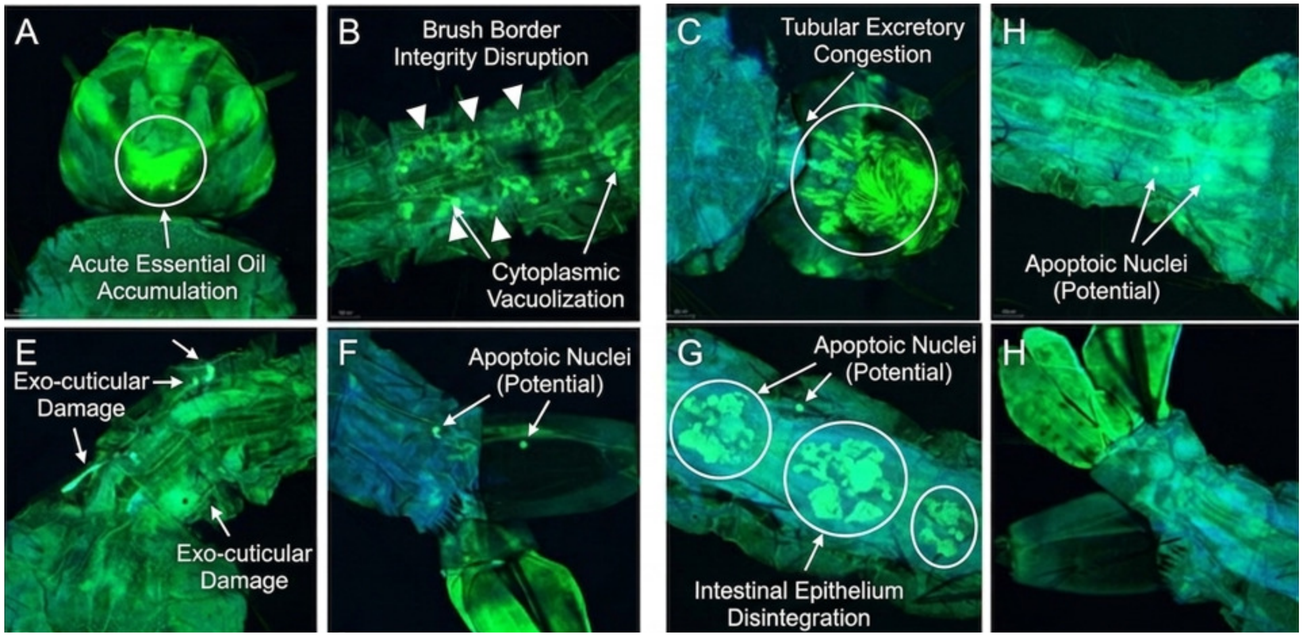

Figure S1. Confocal laser scanning microscopy (CLSM) of *Ae. aegypti* larvae treated with essential oil from *P. humillimum*. A) Cephalic region showing acute accumulation of the essential oil (high fluorescence intensity) in the oral cavity and feeding apparatus. B) Longitudinal section of the midgut exhibiting severe disruption of the brush border integrity, accompanied by extensive cytoplasmic vacuolization (arrowheads), indicating cellular stress. C) Thoracic region highlighting congestion and structural damage to the tubular excretory system. D and F) Posterior segments showing fragmented fluorescence patterns, potentially representing apoptotic nuclei or chromatin condensation. E) Lateral view of the abdomen demonstrating significant exo-cuticular damage and loss of chitinous layer uniformity. G) Detail of the abdominal midgut showing advanced disintegration of the intestinal epithelium and the presence of localized fluorescent clusters. H) Anal papillae and terminal segment displaying altered morphology and irregular distribution of the essential oil within the anal saddle.

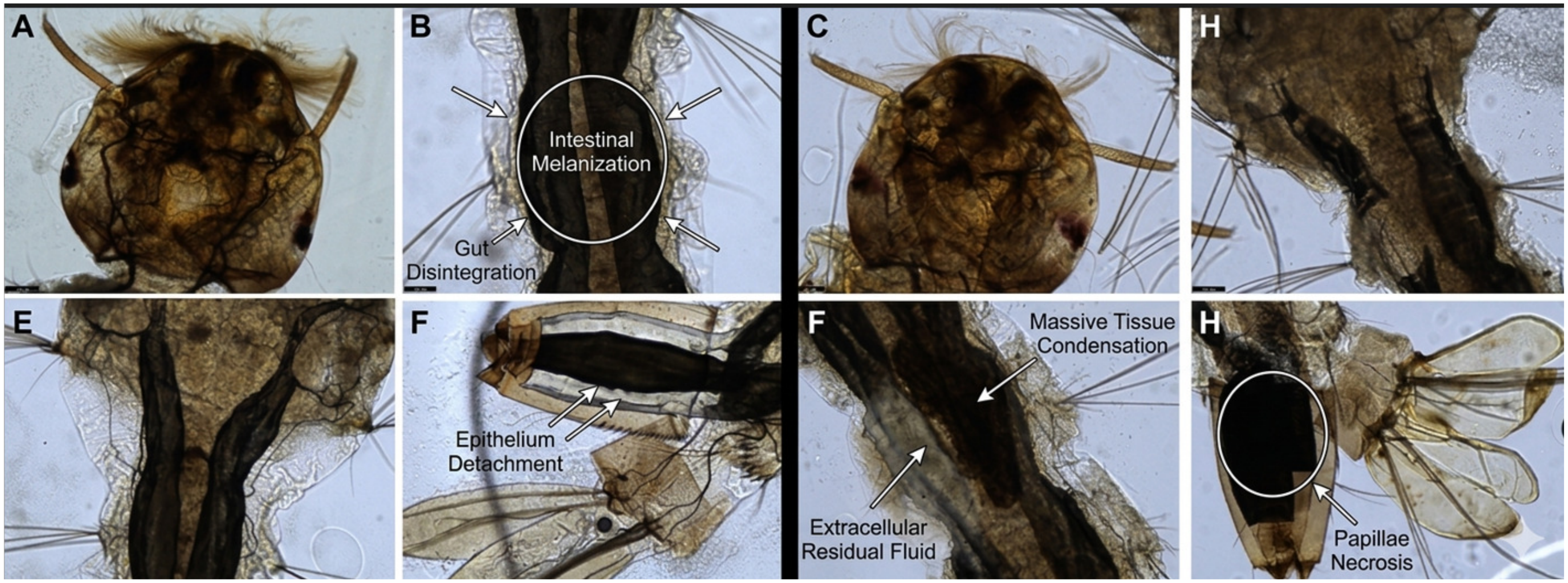

Figure S2. Morphological and histopathological alterations in *Ae. aegypti* larvae observed by light microscopy after exposure to essential oil from *P. humillimum*. (A) Head capsule displaying pronounced internal tissue darkening and loss of structural definition in the cephalic region. (B) Thoracic segment showing severe narrowing of the anterior midgut and initial signs of peritrophic matrix degradation. (C) Detail of the head highlighting localized necrotic foci (dark pigmented spots) near the ocular and feeding apparatus. (D) Prothoracic region exhibiting marked tissue contraction and significant disorganization of the internal architecture. (E) Anterior midgut demonstrating the hallmark of cytotoxicity: extensive detachment of the intestinal epithelium from the basement membrane, resulting in a constricted central canal. (F–G) Posterior abdominal segments displaying massive tissue melanization and darkening along the alimentary canal, indicative of advanced systemic necrosis. (H) Terminal segment and anal papillae showing altered morphology, including cellular swelling and loss of transparency, suggesting a complete collapse of osmoregulatory capacity. Scale bars: 100 μm. The observed darkening, epithelial shedding, and organ deformation collectively indicate severe cytotoxic and neurotoxic effects leading to rapid larval mortality.

In vitro enzymatic assays

Measurement Glutathione S-Transferase (GST) activity

Direct exposure to the treatments resulted in a potent inhibition of GST activity in both species (*Ae. aegypti*: F (4, 10) = 94.04; *An. darlingi*: F (4, 10) = 868.4; $p < 0.0001$). In *Ae. aegypti*, the essential oil (13.33 ± 2.51 μmol min^{-1} mg^{-1} protein), germacrene D (16.00 ± 2.00), and δ-cadinene (12.33 ± 2.08) suppressed enzymatic activity to levels statistically equivalent to the synthetic control α-cypermethrin (6.33 ± 2.51) ($p > 0.05$).

All botanical groups remained significantly lower than the DMSO control (66.67 ± 8.73). A similar magnitude of inhibition was observed for *An. darlingi* (Figure 1), where the botanical treatments reduced activity by approximately 80% compared to the negative control (89.33 ± 2.08), highlighting GST as a direct target for these sesquiterpenes.

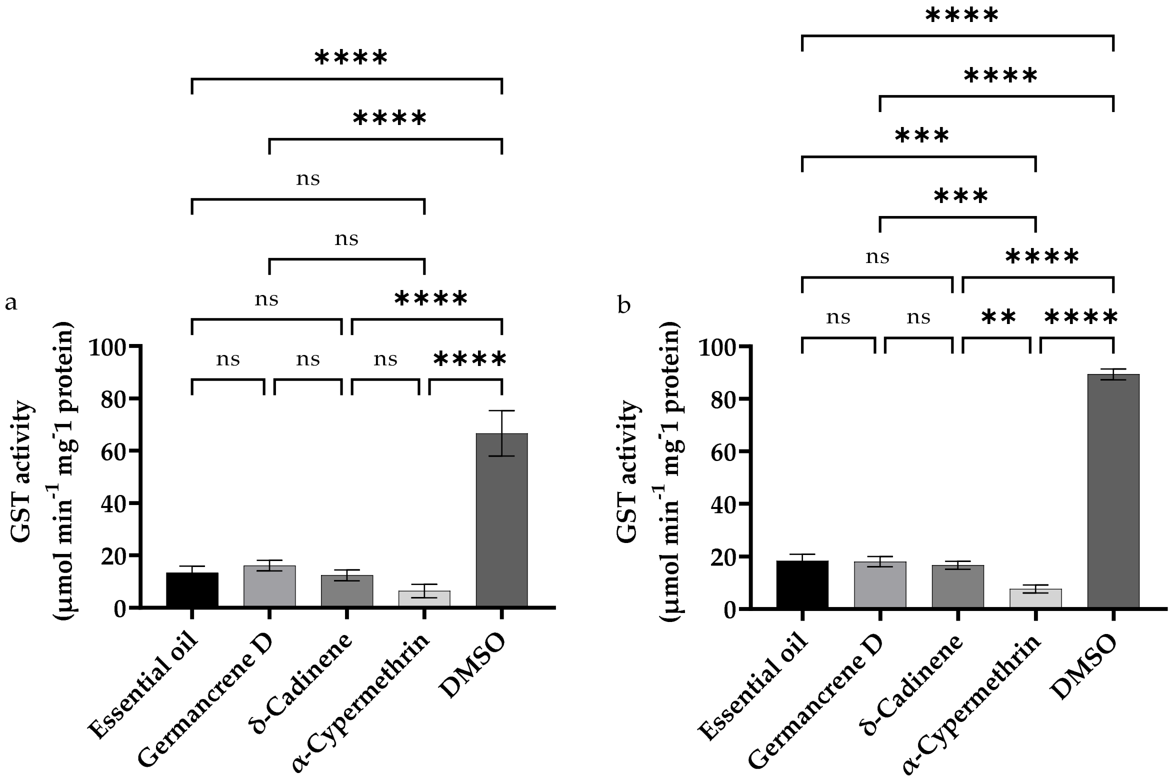

Figure S3. Effects of the essential oil from *P. humillimum* along with germacrene D, δ-cadinene, and α-cypermethrin on GST activity in (a) *Ae. aegypti* and (b) *An. darlingi*. DMSO was employed as the control group are expressed as mean ± standard deviation. Statistical significance is indicated as follows: ** ($p < 0.01$), *** ($p < 0.001$), **** ($p < 0.0001$), and ns (not significant) according to one-way ANOVA followed by Tukey's post-hoc test.

Measurement Mixed-Function Oxidase (MFO) activity

MFO activity showed high susceptibility to the botanical compounds in both species (*Ae. aegypti*: F (4, 10) = 208.9; *An. darlingi*: F (4, 10) = 132.7; $p < 0.0001$). For *Ae. aegypti*, the essential oil and isolated sesquiterpenes triggered an induction nearly 14 times greater than the basal levels of the DMSO group (4.33 ± 1.52 nmol cyt c min^{-1} mg^{-1} protein).

In *An. darlingi*, a remarkably uniform activation pattern emerged; the essential oil (55.67 ± 4.16), germacrene D (58.33 ± 2.08), and δ-cadinene (58.00 ± 6.55) promoted comparable levels of induction ($p > 0.05$). While these activation peaks remained below the α-cypermethrin levels (Figure 2), they confirm that the tested compounds are readily processed by the MFO system.

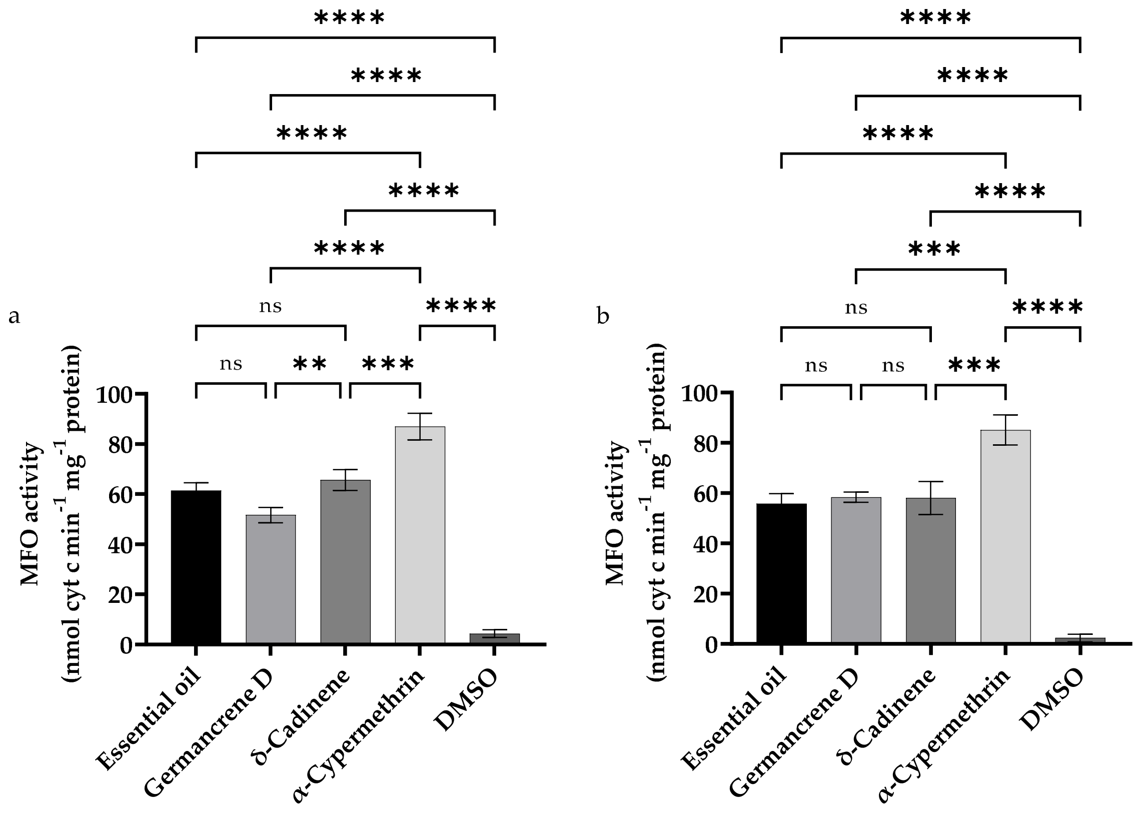

Figure S4 Effects of the essential oil from *P. humillimum* along with germacrene D, δ-cadinene, and α-cypermethrin on MFO activity in (a) *Ae. aegypti* and (b) *An. darlingi*. DMSO was employed as the control group. Data are expressed as mean ± standard deviation. Statistical significance is indicated as follows: ** ($p < 0.01$), *** ($p < 0.001$), **** ($p < 0.0001$), and ns (not significant) according to one-way ANOVA followed by Tukey’s post-hoc test.

Measurement α- and β-esterase activity

The catalytic activity of esterases was markedly enhanced in the presence of the essential oil and its major substances. Statistical analysis indicated significant variation among treatments for both α-esterases (*Ae. aegypti*: F (4, 10) = 128.6; *An. darlingi*: F (4, 10) = 285.7) and β-esterases (*Ae. aegypti*: F (4, 10) = 361.9; *An. darlingi*: F (4, 10) = 168.2; $p < 0.0001$).

In the α-esterase assays, β-cadinene stood out in *An. darlingi* (32.00 ± 2.64 μmol min^{-1} mg^{-1} protein) as a slightly more active substrate than the essential oil (24.67 ± 1.52) (Figure 3). For β-esterases, the response was statistically uniform across both species; for instance, the essential oil and sesquiterpenes in *Ae. aegypti* formed a single statistical group ($p > 0.05$) with activity levels near 31.00 μmol min^{-1} mg^{-1} protein (Figure 4). This shift from the basal activity of the controls (approx. 2.33–5.00) reinforces the affinity of these enzymes for the botanical substrates.

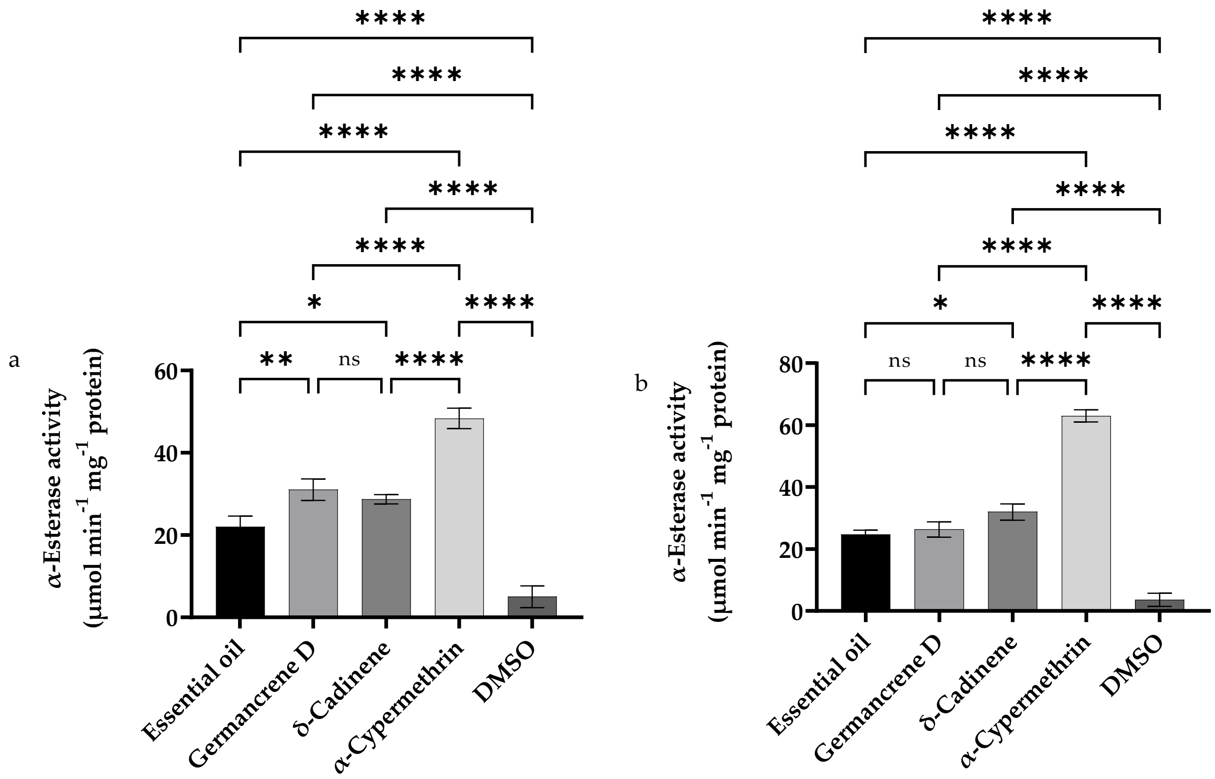

Figure S5 Effects of the essential oil from *P. humillimum* along with germacrene D, δ-cadinene, and α-cypermethrin on α-esterase activity in (a) *Ae. aegypti* and (b) *An. darlingi*. DMSO was employed as the control group. Data are expressed as mean ± standard deviation. Statistical significance is indicated as follows: * ($p < 0.05$), ** ($p < 0.01$), **** ($p < 0.0001$), and ns (not significant) according to one-way ANOVA followed by Tukey's post-hoc test.

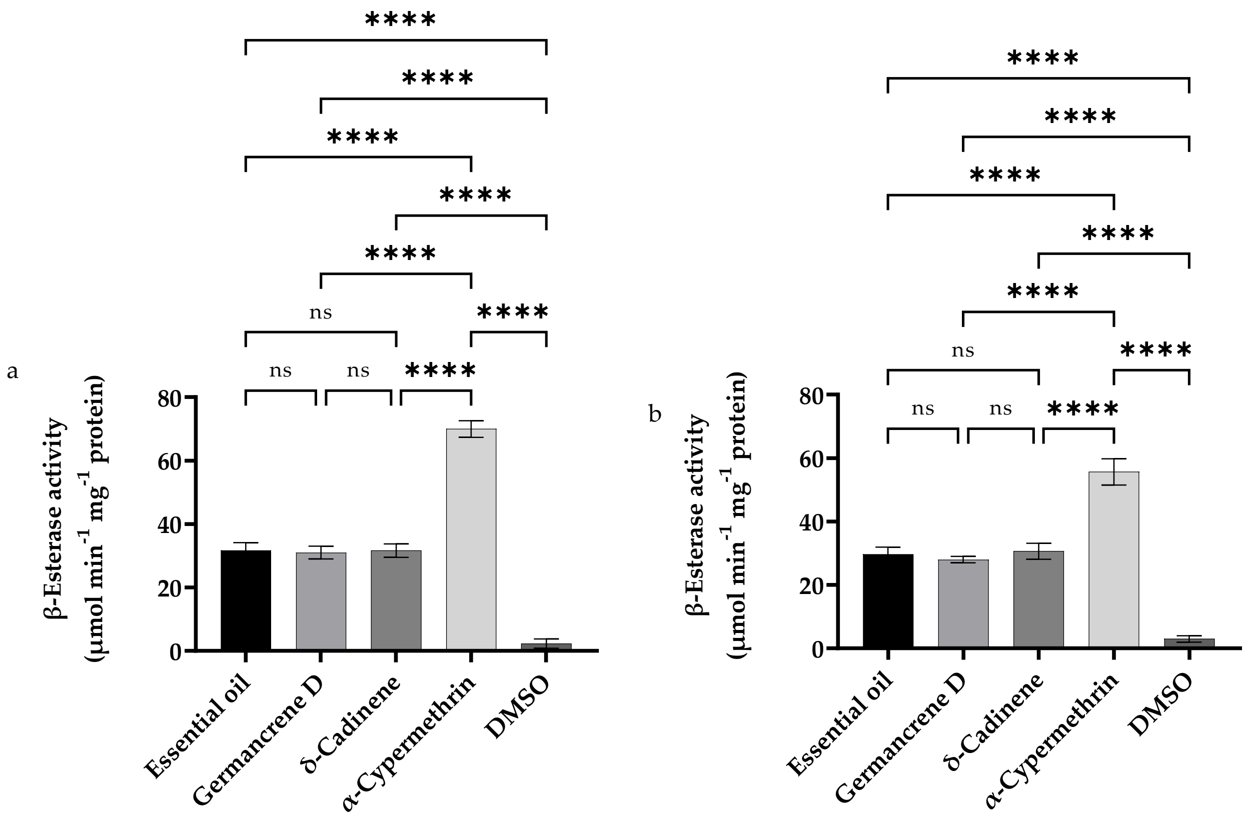

Figure S6. Effects of the essential oil from *P. humillimum* along with germacrene D, δ-cadinene, and α-cypermethrin on β-esterase activity in (a) *Ae. aegypti* and (b) *An. darlingi*. DMSO was employed as the control group. Data are expressed as mean ± standard deviation. Statistical significance is indicated as follows: * ($p < 0.05$), ** ($p < 0.01$), **** ($p < 0.0001$), and ns (not significant) according to one-way ANOVA followed by Tukey's post-hoc test.

Measurement acetylcholinesterase (AChE) activity

AChE was severely inhibited by the botanical treatments in both species (*Ae. aegypti*: F (4, 10) = 148.9; *An. darlingi*: F (4, 10) = 585.6; $p < 0.0001$). In *Ae. aegypti*, the essential oil (13.67 ± 2.51 μmol min^{-1} mg^{-1} protein), germacrene D (18.00 ± 2.64), and δ-cadinene (17.33 ± 2.08) suppressed activity compared to the DMSO control (84.00 ± 8.18).

Notably, the essential oil achieved an inhibitory effect statistically equivalent to α-cypermethrin (7.00 ± 4.00) ($p > 0.05$). For *An. darlingi*, the essential oil (14.33 ± 1.52), germacrene D (18.67 ± 1.52), and δ-cadinene (13.00 ± 3.00) also acted as potent and uniform inhibitors ($p > 0.05$), reducing activity from a basal 90.00 ± 3.60 (Figure 5). These results highlighted AChE inhibition as a key biochemical driver of the larvicidal effect.

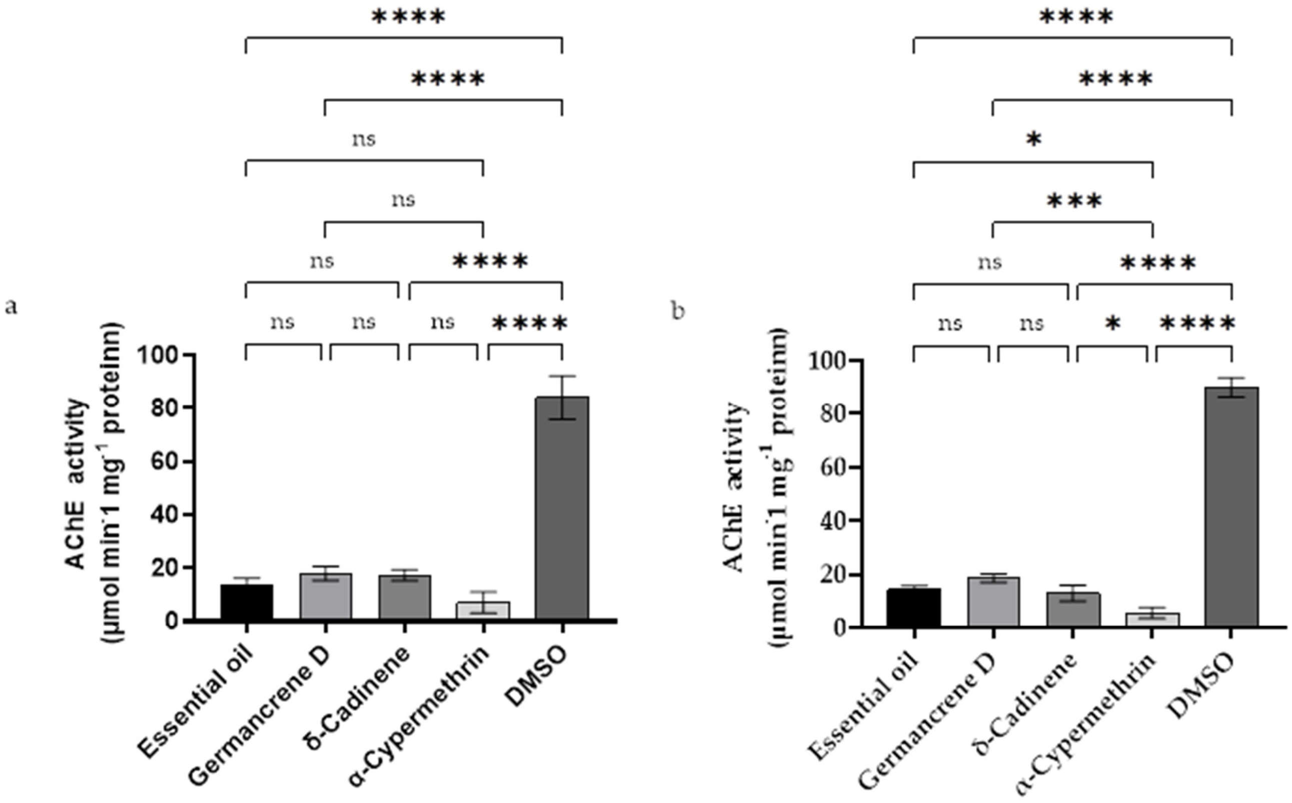

Figure S7. Effects of the essential oil from *P. humillimum*, germacrene D, δ-cadinene, and α-cypermethrin on AChE activity in (a) *Ae. aegypti* and (b) *An. darlingi*. DMSO was employed as the control group. Data are expressed as mean ± standard deviation. Statistical significance is indicated as follows: * ($p < 0.05$), *** ($p < 0.001$), **** ($p < 0.0001$), and ns (not significant) according to one-way ANOVA followed by Tukey's post-hoc test.
